# Exploring ancestral phenotypes and evolutionary development of the mammalian middle ear based on Early Cretaceous Jehol mammals

**DOI:** 10.1093/nsr/nwaa188

**Published:** 2020-08-25

**Authors:** Fangyuan Mao, Cunyu Liu, Morgan Hill Chase, Andrew K Smith, Jin Meng

**Affiliations:** Key Laboratory of Evolutionary Systematics of Vertebrates, Institute of Vertebrate Paleontology and Paleoanthropology, Chinese Academy of Sciences, Beijing 100044, China; CAS Center for Excellence in Life and Paleoenvironment, Beijing 100044, China; Division of Paleontology, American Museum of Natural History, New York, NY 10024, USA; Beipiao Pterosaur Museum of China, Beipiao 122100, China; Microscopy and Imaging Facility, American Museum of Natural History, New York, NY 10024, USA; Microscopy and Imaging Facility, American Museum of Natural History, New York, NY 10024, USA; Division of Paleontology, American Museum of Natural History, New York, NY 10024, USA; Earth and Environmental Sciences, Graduate Center, City University of New York, New York, NY 10016, USA

**Keywords:** Cretaceous, Jehol Biota, malleus-incus, ectotympanic, mammal evolution

## Abstract

We report a new Cretaceous multituberculate mammal with 3D auditory bones preserved. Along with other fossil and extant mammals, the unequivocal auditory bones display features potentially representing ancestral phenotypes of the mammalian middle ear. These phenotypes show that the ectotympanic and the malleus-incus complex changed notably during their retreating from the dentary at various evolutionary stages and suggest convergent evolution of some features to extant mammals. In contrast, the incudomalleolar joint was conservative in having a braced hinge configuration, which narrows the morphological gap between the quadroarticular jaw joint of non-mammalian cynodonts and the incudomalleolar articulations of extant mammals. The saddle-shaped and abutting malleus-incus complexes in therians and monotremes, respectively, could have evolved from the braced hinge joint independently. The evolutionary changes recorded in the Mesozoic mammals are largely consistent with the middle ear morphogenesis during the ontogeny of extant mammals, supporting the relation between evolution and development.

## INTRODUCTION

Attachment of the ectotympanic bone to the otic region and incorporation of the malleus-incus complex in the ossicular chain are two key events in the evolution of the mammalian middle ear; the former holds the tympanic membrane and the latter forms a functionally semi-independent unit that resulted in an increased bandwidth of hearing, particularly of high-frequency sounds [1]. Because the incus (quadrate) is recessed at the periotic in the cranium, it served as the anchor point for the gradual evolutionary shift of the malleus (articular), the gonial (prearticular) and the ectotympanic (angular) away from the dentary bone and its relocation at the base of the cranium [1]. Thus, in addition to understanding the homology of the auditory bones, as reviewed by several authors [2,3], a focused subject in recent paleontological and developmental studies is how these jaw bones were detached from the dentary, which primarily concerns the role played by the Meckel's cartilage and the developmental genetic mechanisms regulating these processes [4–12]. In contrast, the ancestral phenotypes of the mammalian middle ear remain little known due to rareness of fossils. The middle ear of *Didelphis* was considered as the ancestral ear type for therians [1,13], but the saddle-shaped incudomalleolar joint [1,14–17] is already specialized for mammals as a whole. Similarly, the abutting contact of the malleus and incus in monotremes [18,19] is also peculiar [1], even though a similar pattern was claimed to be present in the Mesozoic multituberculate *Jeholbaatar* [20] and the eutriconodontan *Yanoconodon* [7]. A morphological gap exists between the primary quadroarticular synovial jaw joint of non-mammalian cynodonts [21] and the middle ear of extant mammals.

Here we report a new multituberculate mammal from the Early Cretaceous Jehol Biota. The holotype specimen preserves the ectotympanic, malleus-surangular unit, incus and stapes, together with hyoid bones. The 3D morphologies of these elements were revealed by high-resolution CT-scan, which provided the first detailed structures of these auditory bones in multituberculates. Along with those of eutriconodontan *Liaoconodon* [8] and stem therian *Origolestes* [12], by far the most unequivocal middle ears known in Mesozoic mammals, the ancestral phenotypes of the mammalian middle ear can be explored. For comparison, we also present high-resolution 3D reconstructions of the middle ear of *Tachyglossus, Didelphis* and *Erinaceus* as representatives of extant monotremes and therians (marsupials and placentals). Although the latter have been known for many decades, the 3D morphology of the ossicular chain in anatomical articulation has not been portrayed in the way we present in this study.

It has been a common view that the definitive mammalian middle ear (DMME) [22,23] evolved independently in monotremes, therians and multituberculates [1,7,8,12,23]. Although they differ considerably in morphology, the same homologous elements make up the middle ear in monotremes and therians, respectively (the stapes, incus, malleus and ectotympanic). The independent origin of the middle ear in mammals thus only refers to the process and perhaps timing of detachment of the middle ear bones from the dentary [8,9]. Under this assumption we further specify osteological changes of the auditory bones after their detachment in three lineages of Mesozoic mammals, of which the new multituberculate is of particular interest because it provides new morphological evidence of the DMME from an extinct group that is distantly related to monotremes and therians. Our goal is to focus on the transitional morphology that could represent the ancestral phenotypes for mammalian middle ears so that the morphological gap between the mandibular middle ear of non-mammalian cynodonts and the DMME in extant mammals can be bridged. It is also important to have an additional example that shows convergent evolution, thus plasticity in evolutionary development, of the DMME in mammals. Moreover, these ancestral phenotypes provide direct evidence to address the relation of development and evolution in the mammalian middle ear. We show that many, but not all, of the primitive features are recapitulated in embryological morphogenesis of the middle ear in extant mammals and that future researches in paleontology and development biology are needed to answer questions raised in this study.

## RESULTS

### Systematic paleontology

Mammalia Linnaeus, 1758

Multituberculata Cope, 1884

Eobaataridae Kielan-Jaworowska *et al.*, 1987

*Sinobaatar* Hu and Wang, 2002

*Sinobaatar pani* sp. nov.

**Figure 1.**
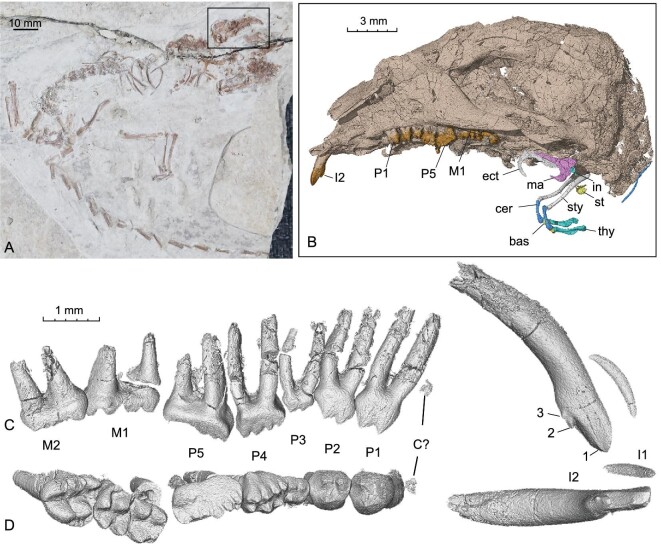
The holotype specimen of *Sinobaatar pani* (BPMC 0051). (A), The holotyp e skeleton. (B), CT-rendered skull corresponding to the boxed area in A, showing reconstructed hyoids and auditory bones. (C, D), Left upper dentition in lingual and occlusal views. Abbreviations: bas, basihyal; C?, upper canine germ?; cer, ceratohyal; ect, ectotympanic; in, incus; M, upper molar; ma, malleus part (surangular not separated); P, upper premolar; st, stapes; sty, stylohyal; thy, thyrohyal. See Supplementary Data.

**Holotype.** The holotype is a disarticulated skeleton (Fig. 1; see Supplementary Fig. 1) (BPMC 0051, Beipiao Pterosaur Museum of China, Beipiao, Liaoning 122100, China).

**Locality and horizon.** Dapingfang, Chaoyang City, Liaoning Province, China; Jiufotang Formation, Early Cretaceous (Aptian) [24,25; see Supplementary Data].

**Etymology.** The specific name is after Junyi Pan, the collector of the holotype specimen.

**Diagnosis.** *Sinobaatar pani* differs from other multituberculates in having the following combination of features: a gracile skeleton with a long tail (possibly arboreal); a strong zygomatic process of the maxilla; a single infraorbital foramen; tooth formula 3-0-5-2/1?-0?-?-2; tooth cusp formula P1–3 (1:2), P4 (3:4), P5 (1:4:2?), M1 (3:4), M2 (Ri:2:3)/m1 (3:2), and m2 (2:2); I2 robust with three cusps (a main mesial one and two minor distal ones); P1–3 cusps showing the trend of coalescing; P3 greatly reduced in size; distal cusp of P4 and mesial one of P5 the highest cusps and forming the peak (in lateral view) in the middle of the cheek tooth row; P5 proportionally not so enlarged relative to P4 and M1; molar cusps slim; distolingual cusp of M1 transversely orientated (mesiodistally short) with labial and lingual ridges; M2 considerably shorter than M1; M2 cusps increasing size distally (see Supplementary Data for detailed description).

### Hyoid apparatus

**Figure 2.**
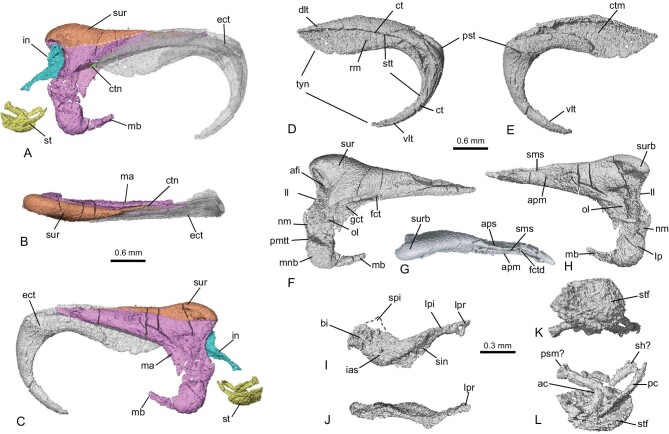
CT rendered auditory bones of *Sinobaatar pani* sp. nov. (A–C), Auditory bones digitally restored in medial, dorsal and lateral views. The dashed line indicates the purported boundary between the fused bodies of the malleus and surangular. The relationship of the stapes to other elements is uncertain. (D, E), Ectotympanic in medial and lateral views. (F-H), Malleus-surangular unit in medial, dorsal and lateral views. (I, J), Incus in lateral and posterior views. (K, L), Stapes in proximal and presumably lateral views. See Supplementary Data. Abbreviations: ac, anterior crus of stapes; afi, articular facet for incus; apm, anterior process of malleus; aps, anterior process of surangular; bi, body of incus; ct, crista tympanica; ctm, contact facet for malleus; ctn, chorda tympani nerve; dlt, dorsal limb of ectotympanic bone; ect, ectotympanic; fct, foramen for chorda tympani; fctd, foramen (exit) for chorda tympani on dorsal side; gct, groove for chorda tympani; ias, incus articular surface for malleus; in, incus; ll, lateral lip of the articular facet; lp, lateral process of malleus; lpi, long (stapedial) process of incus; lpr, lenticular process; ma, malleus part; mb, manubrium of malleus; mnb, manubrial base; nm, neck of malleus; ol, osseous lamina; pc, posterior crus of stapes; pmtt, muscular process for tensor tympani muscle; psm?, process for stapedius muscle?; pst, styliform process of tympanic bone; rm, recessus meatus; sh?, stapedial head?; sin, sulcus incudes; sms, suture between (anterior processes of) malleus and surangular; spi, short process of incus (broken); st, stapes; stf, stapedial footplate; stt, sulcus tympanicus of tympanic bone; sur, surangular part; surb, surangular boss; tyn, tympanic notch; vlt, ventral limb of ectotympanic bone.

*Sinobaatar pani* sp. nov. is represented by a disarticulated skeleton and split skull with some teeth (Fig. 1; see Supplementary Figs 1 and 2, Supplementary Movies 1 and 2). The hyoid apparatus is similar to some eutherians in having seven rod-like elements (the basihyal, ceratohyal, stylohyal and thyrohyal) in which the stylohyal is long and slender (Fig. 1; see Supplementary Fig. 3; Supplementary Movies S3). The epihyal was not preserved; it could be fused with either the ceratohyal or the stylohyal. The auditory bones include both pairs of the mallei and ectotympanic, one stapes and one incus (Figs 1 and 2), which are the focused subject of this report (Figs 1 and 2; see Supplementary Figs 4–9, Supplementary Movies 1,2, 4). Discussions on the auditory bones of *Jeholbaatar* and euharamiyidans are also provided in the Supplementary Data. For convenience of description, we assume that the ectotympanic and malleus were orientated vertically.

### Auditory bones

**Figure 3.**
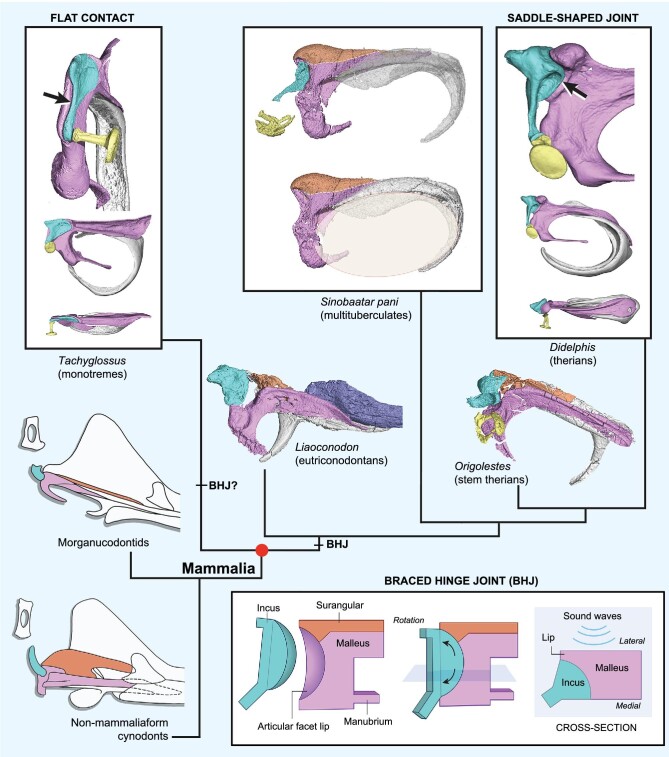
Diagram showing ancestral phenotypes of mammalian middle ear represented by key taxa. The braced hinge joint as an ancestral mammalian condition is illustrated in the cartoon at the lower right, contrasting the quadroarticular jaw articulation in non-mammalian cynodonts and the two types of incudomalleolar joints in therians and monotremes. This joint is present in *S. pani, Liaoconodon* and *Origolestes*. The semi-transparent oval in multituberculate approximates the shape and size of the tympanic membrane. Typical features in key taxa are as follows: **Morganucodontids** (articular, prearticular, angular and surangular attached to the dentary; quadroarticular jaw articulation functional). ***Liaoconodon*** (postdentary bones detached from dentary to form auditory bones but still in contact with ossified Meckel's cartilage; surangular present; braced hinge incudomalleolar on posteromedial end of malleus-surangular unit; long anterior processes of ectotympanic and malleus; dorsal and ventral limbs of ectotympanic short; stapes with broad head and strong process for stapedius muscle (inferred from *Chaoyangodens*)). ***Origolestes*** (bone contact lost between auditory bones and ossified Meckel's cartilage in adult; reduction of anterior limb but more developed ventral and dorsal limbs of ectotympanic; strong process for stapedius muscle of the stapes). ***Sinobaatar pani*** (absence of anterior limb and further developed ventral and dorsal limbs of ectotympanic; tympanic sulcus on entire ectotympanic; development of malleus neck, base and a true manubrium; long process of incus with a narrow end for articulation with the stapedial head). The auditory bones of *S. pani* are distinctly more primitive than those of extant mammals in having a still incomplete ectotympanic, a short manubrium, a braced hinge malleus-incus joint, presence of the surangular that makes the unit heavy, and lack of the bending of the lenticular process. In both *Liaoconodon* and *Origolestes*, the incus was slightly displaced in original preservation so that the articular facet on the malleus can be seen. See Supplementary Data for the middle ears of monotremes and therians.

The ectotympanic is sickle-shaped, consisting of the curved ventral and relatively straight dorsal limbs whose ends are separated by a wide tympanic notch (Fig. 2). The ventral limb, presumably homologous to the reflected lamina of the angular [22], is better developed than that of *Liaoconodon* [8] and *Origolestes* (Fig. 3; see Supplementary Fig. 9) [8], but is less so than the horseshoe-shaped ectotympanic in extant mammals (Fig. 3; see Supplementary Fig. 4) [14,15,17,26]. The dorsal limb is plate-like, uncommon in mammals but reminiscent of the plate-like ectotympanic of *Arboroharamiya* [27,28]; its lateral side bears an extensive contact facet for the malleus, similar to that of monotremes [19]. On the medial side, the crista tympanica is weak on the dorsal limb but distinct in the ventral one so that the tympanic sulcus is shallow on the dorsal limb but deep in the ventral one. The sulcus accommodates the annulus fibrosus, a thickened circumferential rim of the pars tensa of the tympanic membrane [29] that attaches the membrane to the sulcus. The ectotympanic and the malleus form an incomplete oval frame for supporting the tympanic membrane, which gives an estimated area of 4.24 mm^2^ for the membrane.

We interpret that the bodies of the malleus and surangular are fused but their anterior processes are separated by a suture (Fig. 2; see Supplementary Data and Supplementary Fig. 7). The combined malleus-surangular unit is robust compared to the malleus of extant mammals. The surangular part is posterolateral to the malleus, with its anterior process inserting between the ectotympanic and the malleus and gradually tapering anteriorly. On the medial side of the malleus, a groove leads to the foramen for the chorda tympani nerve that pierces the anterior process, echoing the view that the anterior process is homologous to the prearticular in non-mammalian cynodonts [19,30]. The canal does not penetrate the anterior process of the surangular but exits dorsally from a slit between the anterior processes of the surangular and malleus; this serves as an evidence for the identification of the surangular. The articular facet for the incus is a crescent concavity at the posteromedial end of the

 malleus-surangular unit; it is primarily within the malleus and dorsally bounded by the surangular, similar to that in *Liaoconodon* [8] and *Origolestes* (Fig. 3; see Supplementary Fig. 9) [12]. A narrow bony lip extends along the lateral edge of the facet, which braces the articular facet. Between the articular facet and the manubrial base is the neck; its posterior border is shallowly concave so that the posterior border of the malleus shows a double-concavities, similar to that of *Didelphis* [17] and monotremes (Fig. 3; see Supplementary Fig. 4) [19]. The manubrial base thickens notably so that a step-like boundary is formed between it and the neck and the manubrium. Because of this configuration, the manubrial base on the

 

 

lateral side was misidentified as the incus in *Jeholbaatar* [20]. The manubrium is a thin and short prong, parallel to the anterior process and tapering distally.

The incus is proportionally small and quite flat but differs from the platelet-like incus of monotremes (Fig. 3; see Supplementary Fig. 8) [19]. It has a body with a convex articular surface for the malleus, a short process (broken) and a long process that has an angle with the body. The distal end of the long process flares to give the shape of a lenticular process, but the latter does not show a bending from the long process. The stapes has an oval footplate with an estimated area of 0.215 mm^2^; it is convex medially toward the fenestra vestibuli and concave on the lateral surface, similar to the Jurassic multituberculate *Pseudobolodon* [31] and the stem therian *Origolestes* [12]. Judging from the crushed segments, the stapes is most likely bicrural, with the anterior crus being near the center and the posterior one at the edge of the footplate, as in *Pseudobolodon* and *Origolestes*. There should be a sizable stapedial foramen, but a meaningful reconstruction of the complete stapes is difficult.

Based on the frame formed by the ectotympanic and malleus, the estimated area of the tympanic membrane (4.24 mm^2^; Supplementary Data) gives an effective area of 2.827 mm^2^; the transformer ratio between the effective area and the stapedial footplate (0.215 mm^2^) is 13.15, larger than that of *Morganucodon* (10.71) [32]. The tympanic membrane to the stapedial footplate area ratio is 19.7, similar to some neonatal mammals [33,34] but larger than that of *Morganucodon* (16.0); this ratio increases during ontogeny in extant mammals. The transformer ratio of the levers [1,26] is difficult to determine because of the displaced ossicles, but development of the manubrial neck and the stapedial process of the incus would affect the ratio. The transversely narrow ectotympanic is tightly bound with the malleus so that both (plus the surangular in the fossil forms) will vibrate as a unit. Such a unit would have a relatively heavy mass so that these Mesozoic animals could hear only relatively lower frequency airborne sounds in a narrower range of frequency [35], as in monotremes [36]. Interestingly, the inner ear of all these forms has only developed a curved cochlea, perhaps a receiving system (inner ear) that matches the delivering system (middle ear) [37].

## DISCUSSION

Recent paleontological and developmental studies have converged on detachment of the auditory bones from the dentary during the evolution and development of the mammalian middle ear [7,8,10–12]. With the new evidence, we further argue that auditory bone features also reflect the relation between development and evolution and largely endorse that ‘portions of the ossicles that are phylogenetically older develop earlier than portions representing more recent evolutionary inventions’ [38]. These evolutionary changes are best preserved in the 3D auditory bones from Mesozoic representatives of three major mammalian clades: the eutriconodontan *Liaoconodon* [8], the stem therian *Origolestes* [12] and the multituberculate *Sinobaatar pani* sp. nov. reported in this study (Fig. 3; see Supplementary Fig. 9, Supplementary Movies 1–6). We view these forms as representing ancestral phenotypes of the mammalian middle ear at different evolutionary stages. While the auditory bones already detached from the dentary in the three phenotypes, the transitional middle ear of *Liaoconodon* is most primitive in that the malleus and ectotympanic have long anterior processes that are still in contact with the ossified Meckel's cartilage; thus, hearing and chewing functions were not completely separated. *Origolestes* is further derived, as it lost the bony contact of the auditory bones to the ossified Meckel's cartilage so that hearing and chewing functions were decoupled [8]. The definitive mammalian middle ear of *S. pani* is further derived, in having some features similar to those of extant mammals but still more primitive than the latter in several aspects (Fig. 3 caption; see Supplementary Fig. 9); it would not be a surprise if a similar middle ear is found in a basal therian in the phylogenetic tree between *Origolestes* and *Didelphis* or in a species basal to monotremes.

### Evolutionary development

In mammalian ontogeny the developing ectotympanic starts as a tri-pronged structure with an anterior limb; the rest of the ectotympanic forms a partial circle [30,39–42]. This configuration is similar to the ectotympanic of *Liaoconodon*; the latter is the closest approximation of the angular bone of *Morganucodon* [21]. In a sequential way, the anterior limb is resorbed and the dorsal limb (relatively straight) and the ventral one (more curved) gradually elongate and become more complete as a horseshoe-shaped frame with a small tympanic notch in later stages of ossicular development. This trend is well reflected in the gradual evolutionary changes of the ectotympanic in *Origolestes, Sinobaatar* and the Cretaceous eutherians, such as *Uchkudukodon* [43] and *Ambolestes* [44]. The developing ectotympanic is transversely narrow at early stage, but gradually expands laterally to form the bulla or external auditory meatus; the early developmental stage of extant mammals, again, is echoed by the evolutionary pattern of the fossils.

It has been shown that a part of the manubrial base of the malleus, either termed the orbicular apophysis or the processus brevis [13] (see Supplementary Data), is homologous to the retroarticular process of the articular because it arises from the second pharyngeal arch [45]; this finding lends support to the view that the manubrium is a neomorph [23]. The manubrium has been interpreted as being absent in *Liaoconodon* [8] but present in *Origolestes* [12]. In the light of *S. pani*, it is most probable that the manubrial neck and a true manubrium were not yet developed in both *Liaoconodon* and *Origolestes* but did evolve in *S. pani* and *Uchkudukodon. Origolestes* has a short process that tapers anteriorly, similar to *Liaoconodon* and differing from the indentation of the manubrial neck and expansion of the manubrial base in *S. pani*. What previously identified as the manubrium in *Origolestes* appears to be the ventral extension of the malleus. Our data suggest that the manubrium probably evolved along with formation of the manubrial neck; both would increase the lever ratio for sound transmit of the ossicular chain [1,26]. The formation of the manubrium is also coordinated with development of the ectotympanic, regulated by various genes and developmental mechanisms [4]. We infer that the more complete ectotympanic in *S. pani* may have played a role in the evolutionary development of the manubrium. These auditory bones show evolutionary and developmental consistency in detailed morphologies. For instance, in the primitive middle ear of *Liaoconodon* the ectotympanic with a long anterior limb but poorly developed dorsal and ventral ones is associated with the malleus that lacks the manubrium. In the relatively derived middle ear of *S. pani*, however, the ectotympanic without the anterior limb but with better developed dorsal and ventral ones is associated with the malleus that has developed the neck, base and manubrium. Morphologically, the auditory bones in *Origolestes* seem to be intermediate between those of *Liaoconodon* and *S. pani*. These configurations are comparable to the morphogenesis during the embryonic development of the middle ear in extant mammals, such as that the neck and manubrium are developed and ossified in later stages of the malleus and the ectotympanic develops from a tri-pronged form to a horseshoe-shaped frame [30,38–42].

The malleus-surangular unit of *S. pani* adds to the increasing evidence that the surangular did not disappear abruptly during the evolution of the mammalian middle ear but had persisted in basal mammals as a primitive character [8,12,20,27]. In these basal forms the surangular occupies a similar position in relation to the malleus and contributes to the articular facet for the incus (Fig. 3; Supplementary Fig. 9, Supplementary Movies 4–6). Such a pattern is comparable to the quadroarticular articulation of *Morganucodon* in which the surangular forms a considerable part of the articular facet [21]. Its disappearance in extant mammals may be attributed to the evolutionary reduction of the ear ossicle mass for efficient hearing of high-frequency sounds, but whether the surangular survived as a remnant in the embryonic stage of extant mammals remains unclear [3,17,46].

### Braced hinge joint

The incudomalleolar hinge joint differs in shape and position from those of monotremes and therians (Fig. 3; see Supplementary Fig. 4). Nonetheless, it retains the convex-to-concave articulation between the incus (quadrate) and malleus (articular), the basic pattern of the primary synovial jaw joint that persisted throughout the evolutionary radiation of the mammalian malleus-incus complex, except for monotremes [1]. Functionally, because the lateral lip braced the articular facet, airborne sounds coming from the lateral side of the tympanic membrane could be efficiently transmitted to the incus and stapes; at the same time, the crescent facet allows some rotation of the incus relative to the malleus (Fig. 3). This joint appears to be a conservative feature present in at least the three Mesozoic mammals discussed in this study but has not been documented in known embryonic morphogenesis of extant mammals [38,40,41]. Because the incus anchors at the periotic in the cranium, roughly retaining the position of its precursor (quadrate) in non-mammalian cynodonts; the gradual shift of the malleus (articular), gonial (prearticular) and ectotympanic (angular) was basically a rearward retreat from the dentary during the evolution of mammals [1]; thus, changes and reorientation of the auditory bones were more significant at their anterior and ventral side than at the incudomalleolar articulation. In contrast to the conservative braced hinge joint, these changes suggest that developmental heterochrony has played a role in the evolutionary development of the mammalian middle ear. Given its shape and composition, the braced hinge joint is derivable from the quadroarticular jaw articulation in non-mammalian cynodont. On the other hand, the saddle-shape joint of therians [1,14,15,17,26] could be derived from the braced hinge joint by shift of the incus to the caudal side of the malleus. Still, it remains unclear how the abutting condition in monotremes evolved because the phylogenetic components and middle ear fossils in the lineage toward monotremes are poorly known. It is possible that the monotreme condition may have also derived independently from a similar braced hinge pattern by migration of the incus to the dorsal side of the malleus. Developmental study may prove to be indicative for this issue, given that new observations continue to become available, such as that the ectotympanic and malleus of the echidna are originally in a vertical position in early ontogeny, similar to therians, before flipping to the horizontal condition in the adult [42].

### Ancestral phenotypes

With the assumption that the DMME evolved independently in monotremes, therians and multituberculates [1,7,8,12,23,27], there should be no common ancestral phenotype of the middle ear for these clades. However, the auditory bones of *Liaoconodon, Origolestes* and *S. pani* display some shared primitive features, such as the braced hinge incudomalleolar joint, an incomplete ectotympanic, and presence of the surangular, that potentially illustrate the ancestral phenotype of the mammalian middle ear in each lineage. These forms narrow the morphological gap between the mandibular middle ear of non-mammalian cynodonts and the DMME of extant mammals. The differences of these phenotypes, such as the degree of development of the ectotympanic (its anterior, dorsal and ventral limbs), the morphology of the malleus (development, or not, of the manubrial neck, base and the manubrium), and the fusion or separation of the malleus and the surangular, are interpreted as representing various evolutionary stages in different lineages. These phenotypes show comparable pattern with the morphogenesis of the middle ear in extant mammals and to some degree support the relation between evolution and development. The derived features of *S. pani*, such as development of the manubrium, must be interpreted as a result of convergent evolution to those of extant mammals, which suggests plasticity in the evolutionary development of the middle ear; it illustrates that evolutionary ‘experiments’ for better hearing had taken place in various clades during mammalian evolution. Future paleontological and developmental studies are needed to test the issues raised by the discoveries of the Mesozoic mammals, as we presented in the study.

## MATERIALS AND METHODS

### Specimens and provenance

The holotype specimen of *Sinobaatar pani* sp. nov. (BPMC 0051, Beipiao Pterosaur Museum of China, Fig. 1; see Supplementary Fig. 1) is a disarticulated skeleton, with the skull split into two parts; some teeth and most hyoid and auditory bones were well-preserved within the matrix and were revealed by CT scan (see Supplementary Figs 2,3,5–8). The specimen was collected by Mr. Junyi Pan with one of the authors (C.L.) on site from the Early Cretaceous Jiufotang Formation at Dapingfang, Chaoyang City, Liaoning Province, China. The specimen is under C.L.’s curation and deposited at the Beipiao Pterosaur Museum of China, Beipiao County, Liaoning Province, China. The specimen will be accessible for researchers after its publication. The digital data, which is the primary data for this study, will be available upon request for research purposes.

Several specimens are used for comparison. The CT-rendered middle ear of *Liaoconodon* from the holotype (IVPP V16051) [8] represents previously unpublished data. The CT-rendered middle ear of *Origolestes lii* was based on the paratype specimen (JZT-DB0064) [12]. The provenance of these specimens was given in the original studies. The middle ear bones of *Didelphis* and *Tachyglossus* were based on articulated ossicles from specimens in the teaching collection of the Division of Paleontology, American Museum of Natural History (AMNH), New York; those of *Erinaceus* are from the Department of Mammalogy, AMNH.

### Imaging

Optical images were taken using a Canon Digital camera with a macro lens installed in the Key Laboratory of Vertebrate Evolution and Human Origins, Institute of Vertebrate Paleontology and Paleoanthropology (IVPP), Chinese Academy of Sciences (CAS).

X-ray microcomputed tomography and imagery were conducted using different methods given the different preservation and size of the specimens. The specimens were preserved in slabs so that the holotype specimens of *Sinobaatar pani* sp. nov. and *Liaoconodon* were first scanned using a micro-computed laminography (CL) scanner in the lab at the IVPP (developed by the Institute of High Energy Physics, CAS). The specimen was scanned with a beam energy of 60–90 kV and a flux of 40–80 μA at a resolution of 6.47–60.57 μm per pixel using a 360° rotation with a step size of 1°. A total of 360 image slices with a size of 2048 by 2048 were reconstructed using a modified Feldkamp algorithm developed by the Institute of High Energy Physics, CAS.

The subsequent high-resolution micro-CT scanning of *S. pani* (BPMC 0051), *Origolestes* (JZT-DB0064) and *Liaoconodon* (IVPP V16051) were conducted using a GE v|tome|x m dual tube 240/180 kV system in Yinghua Inspection and Testing (Shanghai) Co., Ltd. Supplementary Fig. 5A and B shows the examples of the scan results of the holotype of *Sinobaatar pani* sp. nov., which are primary data on which the bones were reconstructed. The specimens of extant *Didelphis, Tachuglossus* and *Erinaceus* were scanned using a GE v|tome|x s 240 dual tube 240/180 kV system (General Electric, Fairfield, CT, USA) in the Microscopy and Imaging Facility of the AMNH. Skull specimens were re-scanned using the 240 kV microfocus tube at 5–15 microns/voxel resolution, 100–160 kV and 100–60 μA. Where needed, a 0.1 mm Cu filter was used to reduce beam hardening artifacts. To improve the signal-to-noise ratio, 1800 projections were collected, for 333–2000 ms and averaged 2–3 times. To accommodate the length of the specimen, 2–4 total areas were scanned in the Y-axis (multiscan) to produce the final projection stack that was reconstructed using Phoenix datos|x (General Electric, Wunstorf, Germany). All of the segmentation and the rendering of the CT scanning data were processed using VGStudio Max 3.1 (Volume Graphics, Heidelberg, Germany).

## DATA AND MATERIALS AVAILABILITY

All data are available in the manuscript or the supplementary materials. The Life Science Identifier (LSID) for the new species has been deposited at ZooBank: LSIDurn:lsid:zoobank.org: act:2C79CD98-F836-4D9A-B788-25676BD1808B.

## Supplementary Material

nwaa188_Supplemental_File
