# Supplementary material for: Exploring ancestral phenotypes and evolutionary development of the mammalian middle ear based on Early Cretaceous Jehol mammals
Source: Natl Sci Rev. 2020 Aug 25;8(5):nwaa188. doi: 10.1093/nsr/nwaa188 (PMC8288399; doi:10.1093/nsr/nwaa188)
Supplement: nwaa188_Supplemental_File [file nwaa188_supplemental_file.zip › NSR_2020-778-supplementary data.docx]

Supplementary Data for

**Exploring ancestral phenotypes and evolutionary development of the mammalian middle ear based on Early Cretaceous Jehol mammals**

Fangyuan Mao^1,2,3^*, Cunyu Liu^4^, Morgan Hill Chase^5^, Andrew K. Smith^5^, Jin Meng^3, 6^*

Correspondence to: [jmeng@amnh.org](mailto:jmeng@amnh.org) (J. M.); [maofangyuan@ivpp.ac.cn](mailto:maofangyuan@ivpp.ac.cn) (F. M.).

**This PDF file includes:**

Materials and Methods

Supplementary Text

References

Supplementary Figure 1. The holotype specimen of *Sinobaatar pani* sp. nov. (BPMC 0051).

Supplementary Figure 2. CT-rendered dentitions of *Sinobaatar pani* sp. nov. (BPMC 0051).

Supplementary Figure 3. Digital rendered hyoid apparatus of *Sinobaatar pani* sp. nov*.* (BPMC 0051).

Supplementary Figure 4. CT-rendered auditory bones of monotremes (*Tachyglossus*), marsupials (*Didelphis*), and placentals (*Erinaceus*).

Supplementary Figure 5. Preservation and auditory bones of *Sinobaatar pani* sp. nov. (BPMC 0051).

Supplementary Figure 6. Ectotympanic (tympanic) bone of *Sinobaatar pani* sp. nov. (BPMC 0051).

Supplementary Figure 7. Malleus and surangular of *Sinobaatar pani* sp. nov*.* (BPMC 0051).

Supplementary Figure 8. Incus and stapes of *Sinobaatar pani* sp. nov. (BPMC 0051).

Supplementary Figure 9. Incus and stapes of *Sinobaatar pani* sp. nov. (BPMC 0051).

**Other Supplementary Materials for this manuscript include the following:**

Supplementary Movie 1. Preservation of the skull and auditory bones of *Sinobaatar pani* sp. nov.

Supplementary Movie 2. Reconstructed hyoids and auditory bones.

Supplementary Movie 3. Hyoid apparatus of *Sinobaatar pani* sp. nov*.*

Supplementary Movie 4. Auditory bones of *Sinobaatar pani* sp. nov.

Supplementary Movie 5. Auditory bones of *Liaoconodon*.

Supplementary Movie 6. Auditory bones of *Origolestes*.

**Materials and Methods**

Specimen Preparation

The holotype skeleton was preserved in the matrix of one slab and partly exposed to show the general shape and some details of the skeleton. Because of the gracile skeleton and the broken skull, further mechanical preparation was difficult or in some areas impossible (such as the miniscule auditory bones embedded in the matrix). After the initial preparation and CT-scan of the skeleton, the slab containing the left skull was separated along the crack for a higher resolution CT-scanning, as shown in Fig. 1 and Supplementary Fig. 1. No destructive method was applied to the fossil during the preparation process.

Measurements

Because the fossil is partly in the matrix, particularly the ear bones and teeth, measurements were taken using digital methods from the CT images. After these elements were segmented out and reconstructed, the linear measurement was firstly taken using the Measurements Menu/Coordinate Measurement module in VG Studio Max 3.1 and rechecked with the results using Straight selection in ImageJ 1.49v. The area of tympanic membrane and stapedial footplate were measured using Freehand selections in ImageJ 1.49v, as shown in the figure below in which the measured area is surrounded by the yellow line. All measurements were independently recorded by two authors (F.M. and J.M.) and then averaged to minimize potential bias. The measurements of teeth are in Supplementary Fig. 2 caption, whereas those of the hyoids are in Supplementary Fig. 3 caption.

There are two ratios between the areas of the tympanic membrane and the footplate of the stapes. The first one is the effective area of the tympanic membrane to the footplate area. The effective area is two-thirds of the total area of the tympanic membrane, which concerns the efficiency of transmitting sounds of the membrane so that it is one of the two transformer ratios (the other one is the transformer ratio of the levers, derived from the ear bone structures). The second one is the total area of the tympanic membrane to the footplate area. This ratio gradually increases during embryonic development in mammals. These numbers from the same individual are first known in multituberculates to our knowledge (the footplate area may be estimated from the area of the oval window) and are potentially useful to infer hearing ability and evolutionary development of the middle ear (see references [32,33,34] in the main text).


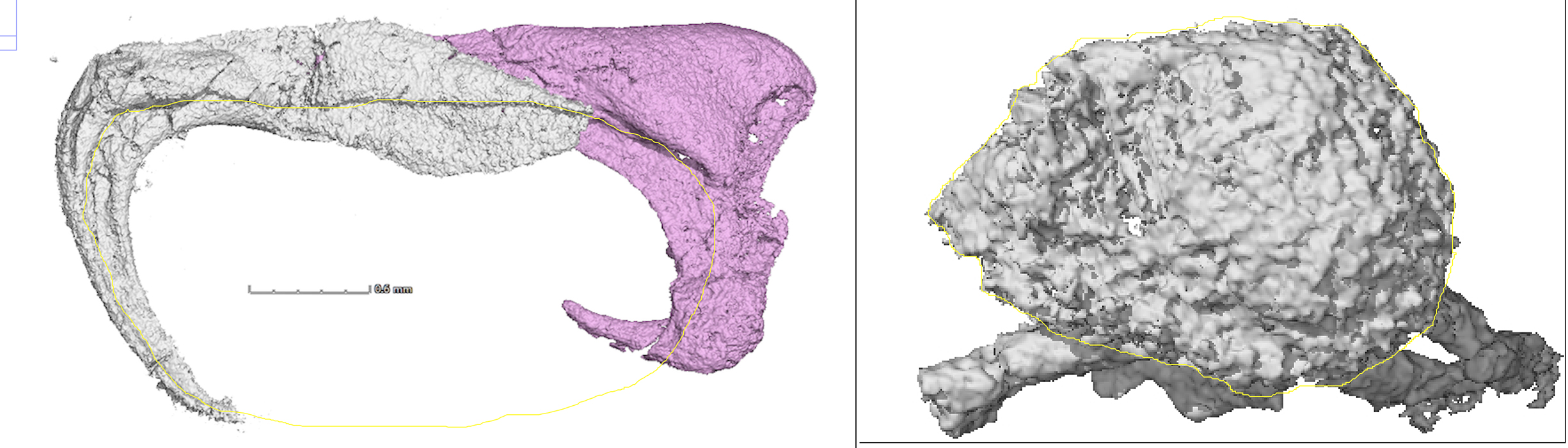


Phylogeny

Given several recent efforts of higher-level phylogenies of mammaliaforms [1-3] and the relatively stable position of multituberculates (*Sinobaatar* in particular) within mammals in all these studies, we consider it redundant to run another phylogenetic analysis. We use a simplified phylogenetic frame (Fig. 3) that reflects the consensus of most existing phylogenetical hypotheses of mammaliaforms for illustrating the evolutionary distributions of the auditory bones discussed in the study. Alternative hypotheses did exist. For instence, a recent work shows an interesting result in placing the eutriconodontans outside of mammals [4].

Orientation

Both mallei and ectotympanic bones are preserved, but only one stapes and one incus are revealed by CT-scan (Supplementary Fig. 3). Because these bones are displaced, identification of a bone as a left or right one, its orientations (dorsal or ventral side), and its relationship with other bones are subject to interpretation. Our interpretation is based on the bone morphologies of the new specimen as well as comparison with those of other multituberculates [1,2,5,6], monotremes [7,8] and other mammals where the auditory bones are known, such as *Liaoconodon* [9] and *Origolestes* (Fig. 3; Supplementary Fig. 9)[3]. Unlike *Liaoconodon* and *Origolestes* in which the ossified Meckel’s cartilage (OMC) still exists and the auditory bones were probably orientated in a more or less vertical position, all multituberculates do not have the OMC and thus lack the Meckelian groove on the dentary. It is likely that the auditory bones were supported by cranial structures and positioned more or less horizontally. However, for convenience of description, we assume the malleus and ectotympanic were in a vertical position, which is comparable to those of *Morganucodon*, *Liaoconoddon*, *Origolestes*, and *Didelphis*. Thus, the views of these bones would be referred to as lateral and medial, instead of ventral and dorsal. It should be kept in mind that the lateral and medial sides are corresponding to the ventral and dorsal sides, respectively, of the auditory bones in monotremes because the latter are horizontal in anatomical position [8,10,11].

Terminology

The terminology for the structures of the auditory bones has not been consistently used and we primarily follow Henson [12] and Wible and Spaulding [13] for the terminology of auditory bone structures. However, for the ectotympanic we will use “dorsal limb” (=anterior leg, Henson, [12]) for the part that is in contact with the anterior process of the malleus and “ventral limb” (=posterior leg, Henson, [12]) for the free part that is presumably homologous to the reflected lamina of the angular in non-mammalian cynodonts. This is because in some Mesozoic forms, such as *Liaoconodon* [9] and *Origolestes* [3], the ectotympanic is often tri-pronged, in which an anterior limb (process) is present and extends anteriorly in parallel with the anterior process of the malleus (Fig. 3; Supplementary Fig. 9). In some developmental studies of marsupials, it is shown that the ectotympanic at its earliest embryological stage is a tri-pronged bone with a “horizontal limb” or “anterior process” [14-19].

**Supplementary Text**

Comments on taxonomy

Two multituberculate genera, *Sinobaatar* and *Jeholbaatar*, were reported from the Early Cretaceous Jehol Biota; the former also persisted in the late Early Cretaceous Fuxin Biota [20]. *Sinobaatar lingyuanensis* [21] was from the Yixian Formation, representing the oldest species of the genus, whereas *S. xiei* and *S. fuxinensis* from the Fuxin Biota [22] represent the youngest species of the genus. The taxonomic assignments at the generic level are subjective and given the considerable variations of morphology and temporal distributions, we choose a conservative way to name a new species within the more inclusive genus *Sinobaatar* but note that the new taxon may represent a new genus. There are other differences that we did not include in the diagnosis. For instance, the hyoids of *Sinobaatar* sp. [1] are considerably different from those of *Sinobaatar pani* (Supplementary Fig. 3). Similarly, the ear ossicles of *Jeholbaatar* differ in many aspects from those of *Sinobaatar* *pani* (Fig. 2). These differences may be resulted from different preservations and/or interpretations, but because of their poor fossil records in general, it is not so useful to include these structures in the diagnosis for the new species.

The major issue we confronted is the comparison with *Jeholbaatar*. The holotype of *Jehobaatar* also came from the Jiufotang Formation in a different site. It was claimed that the auditory bones were completely preserved in this taxon [2]. The auditory bones in the holotype specimen of *Jeholbaatar* were known from one side and the CL-scan did not provide convincing 3D images. In the light of the new specimen reported here, it is clear that some identifications of the auditory bones in *Jeholbaatar* were equivocal or incorrect. First, the auditory bones preserved on the right side of the skull were identified as “left middle ear bones exposed in dorsal view” (Wang et al. [2]: fig. 2). It would be difficult to explain why the left auditory ear bones were preserved on the right side of the skull given that the specimen does not seem to be significantly disturbed in preservation (the skull and postcranial). In our view, it is most probable that the ear bones of *Jeholbaatar* are from the right side and exposed in the ventral (lateral) view given its similarity to the lateral morphology of the malleus and ectotympanic of *Sinobaatar pani* reported here and that of *Sinobaatar* sp. [1]. As in *S. pani*, the surangular boss bends laterally (ventrally) and the tympanic sulcus is not visible in the lateral (ventral) view.

One reason that the auditory ear bones of *Jeholbaatar* were identified as exposed in dorsal view is because of the structure identified as the “incus” and its comparison with that of monotremes. In monotremes, the incus as a platelet is abutted to the dorsal side of the malleus (Supplementary Fig. 4)[8]. However, our new specimen shows that the structure identified as the “incus” in *Jeholbaatar* is unquestionably the base of the manubrium (Fig. 2; Supplementary Fig. 7); it is distinctively thicker than the manubrium in both the dorsal and ventral views, but more so in the ventral view so that it appears as if it was a separate element if only the ventral (lateral) view is available. Moreover, although the auditory bones were reported as “articulated nearly in anatomical position”, the incus was moved to a position closer to the surangular boss in the reconstructed middle ear (Wang et al. [2]: fig. 2d) without explanation. The malleus and ectotympanic of *Jeholbaatar* are similar in general morphologies to those of *Sinobaatar* sp. [1] and *S. pani*, but differ in detail, such as the position of the foramen for the chorda tympani nerve and the anterior process of the malleus. The “stapes” of *Jeholbaatar*, with an unproportionally thick columnar shaft and very small footplate, is also distinctively different from that of *S. pani*. We think the incus and stapes of *Jeholbaatar* remain unknown. Because of the misinterpretation of the incus, the “abutting contact” between the malleus and incus, claimed to be similar to that of monotremes, was not supported by evidence. In short, the available data of *Jeholbaatar* are insufficient for an accurate reconstruction of the middle ear, and we think the middle ear of *Jeholbaatar* would be similar to what we reconstructed for *S. pani* given the close phylogenetic relationship and similarities in general skeletal morphologies of the two genera.

Comments on locality and age

The Jehol Biota consists of organisms from both Yixian (below) and Jiufotang (above) formations [22,23]. The chronological durations of the formations and the radio-isotopic ages of the fossil bearing beds have remained controversial for years [24,25]. The Yixian Formation may have spanned an interval of seven million years based on the dates of 129.7±0.5 for a basaltic lava from the bottom of the Yixian Formation and 122.1±0.3 Ma for a tuff from the lowermost part of the overlying Jiufotang Formation (two meters above the unconformity between the two lithological units)[25]. Whether the date 110.59 ± 0.52Ma [26] can be used as the upper limit for the Jiufotang Formation remains uncertain because of the difficulty of the stratigraphic correlation between these sites [25,27]. Other dates include one from the upper part of the Yixian Formation, the Jingangshan beds, which was 126.5 Ma [28]. In addition, two basaltic samples from the Lujiatun beds, previously regarded as the basal part of the Yixian Formation, yielded ages of 125.8 ± 1.0 Ma and 126.0 ± 0.8 Ma, respectively. Thus, the Yixian Formation represents an interval of about 7 Ma from the early Barremian to probably the early Aptian. Regardless of its age duration, the Jiufotang Formation is most likely within the Aptian and possibly extends into the early Albian; it is stratigraphically higher and chronologically younger than the Yixian Formation in western Liaoning and its vicinities. When a specific age has to be given, it was often cited as 120 Ma [9].

In addition to the radio-isotopic dating data, the bio-assemblages from the Jiufotang Formation may be distinguished from that of the Yixian Formation, while both assemblages are from the Jehol Biota [22,23]. Fossils from the Jiufotang Formation include diverse plants, insects, and vertebrates [9,29-31] and is characterized by the fish *Jinanichthys*, differing from the Yixian assemblage that is dominated by *Lycoptera* [32]. Other vertebrates include *Cathayornis*, *Jeholornis*, *Microraptor* [33,34] and a diverse pterosaur fauna [35]. Mammals reported from the Jiufotang Formation include the eutriconodontan *Liaoconodon* [9] and the multituberculate *Jeholbaatar* [2]. The holotype of *Jeholbaatar* was from the Changzigou site of the Sanjiazi township, Lingyuan City, over 100 kilometers away from the type locality of *Sinobaatar pani*, whereas the holotype of *Liaoconodon* was from Xiaotaizi, Lamadong, Jianchang, Liaoning Province; both holotypes (*Jeholbaatar* and *Lioconodon*) are skeletons that are in peach black, suggesting a preservation condition different from that of *S*. *pani*. Another multituberculate species from the Jehol Biota, *Sinobaatar lingyuanensis*, was from the Yixian Formation at Dawangzhangzi site, Lingyuan City, Liaoning Province [21]. Two other multituberculate species of *Sinobaatar*, *Sinobaatar xiei* and *S. fuxinensis*, came from the late Early Cretaceous Fuxin and Shahai formations in Fuxin city and its vicinity, Liaoning [20]; these strata and bio-assemblages are stratigraphically above the Jiufotang Formation.

Detailed Descriptions

**Dentition:** There are three upper incisors but only I1 and I2 were identified; the existence of I3 was interpreted based on its alveolus (Supplementary Fig. 1). The upper tooth formula can be confidently determined as I3-C0-P5-M2. I1 is extremely small, single-rooted and mesial to I2; it tapers toward the crown tip and the root. I2 is robust with a long crown extending mesioventrally. The crown has a main cusp (cusp 1 in Supplementary Fig. 2A2) that is transversely compressed; its medial surface is flatter than the labial one. Distal to the main cusp are two minor cusps, of which the mesial one is larger than the distal one (cusps 2 and 3 in Supplementary Fig. 2A2). The root is long, strong, and gently curved with circular cross-section; it gradually thins to its open end. A short diastema is between the I2 and I3. The I3 alveolus is shallow, suggesting that the root of I3 is short. In ventral view, the outline of the alveolus is transversely narrower than the mesiodistal length. If this was not due to preservation, then I3 would be different from that of *Sinobaatar* and *Jeholbaatar* in which I3 is transversely wider than mesiodistally long.

There is no sign of the upper canine from the external surface of the maxilla, but the CT scan revealed a possible small tooth germ within the bone (Supplementary Fig. 2). At this stage of ontogeny, e.g., all teeth have erupted, if this was a tooth germ, it would unlikely erupt in life. It could, however, represent a remnant upper canine germ that was lost during the evolution of multituberculates.

All upper premolars are preserved in situ, have two roots, and show an S-shaped outline of the occlusal surface in lingual or labial view. P1-3 are similar in having cusp formula 1:2. Differing from *Sinobaatar lingyuanensis* where the cusps are conical and isolated [20,21], the cusps of P1-3 in *Sinobaatar pani* show a trend of coalescence so that the cusps are not so distinctive. P1 and P2 are subequal in size (P1 being slightly larger) and similar in cusp shapes and arrangement; the cusps are ornamented with small radiating enamel ridges and there is no small cuspule. P3 is the smallest and lowest (or deepest dorsally in the occlusal line in labial view) cheek tooth and is much smaller than P1-2. Of the three main cusps, the labial one is more posteriorly positioned (in relation to the lingual cusps) than that of P1-2. The two weak roots are more open and curved. The small P3 suggests that during the evolution of multituberculates, cheek teeth reduction may start from P3.

P4-5 differ from P1-3 in general crown morphology. P4 has a cusp formula 3:4 so that its occlusal outline is more or less rectangular. The lingual cusps show a gradient of size: the mesial cusp is the lowest and smallest and the cusps increase size distally. The cusps increase height distally and the distal cusp is nearly of the same height of the mesial cusp of P5; the two cusps form the “peak” of the second S-shaped wave in the premolar series. Of the three labial cusps, the middle one is the most robust; all of them, particularly the distal two, are distinctively lower than the lingual cusps. P5 is longer than P3, but compared to that of *Jeholbaatar* [2], *Sinobaatar xiei* and *S. fuxinensis* [20], it is relatively transversely narrower and shorter. The cusp formula is 1:3 (plus Ri):2?. One the mesiolabial corner of the tooth crown, there is a small but distinct cusp. Similar to *Jeholbaatar*, *S*. *xiei* and *S. fuxinensis*, P5 has a blade-like main crest extending obliquely from the mesiolingual corner to the distolabial corner of the tooth crown. The anterior half of the crest consists of three cusps and the distal half is ridge-like, although weak cusps are vaguely discernible. On the distolingual side of the main crest, there is a shelf that seems formed by two cusps. P5 differs from that of *Jeholbaatar*, *S*. *xiei* or *S. fuxinensis* in having the mesial cusp the tallest, whereas in the latter two taxa, it is the third cusp that is the tallest, which forms the peak of the S-shaped wave in the premolar series.

M1 is rectangular in occlusal view and has a cusp formula 3:4, differing from that of *Jeholbaatar*, *S*. *xiei*, and *S. fuxinensis*. On the labial cusp row, the mesial cusp is the largest one, and mesial to it there is a cingulum-like extension, which is cannot be counted as a cusp. On the lingual cusp row, cusps are subequal in size. All the M1 cusps are well separated. The lingual cusps do not show notable size differences (usually larger) from those of the labial cusps, which differs from other multituberculates, such as *Jeholbaatar*, *S*. *xiei* and *S. fuxinensis.* A unique feature is the distal lingual cusp is transversely orientated and mesiodistally short; it extends with a labial and a lingual crest. M2 has a cusp formula of Ri:2:3 and is slightly lingually offset as preserved, but it should be certain that it is one cusp row lingual to M1, as in other multituberculates. Unlike that of *Jeholbaatar*, *S*. *xiei* and *S. fuxinensis,* M2 of *S*. *pani* is notably shorter than M1 and has a wider mesial end and a narrow and rounded distal end. There is a cingulum on the mesiolabial side of the crown but no cusp is developed. Of the two labial cusps, the distal one is larger, differing from that of *Jeholbaatar*, *S*. *xiei* and *S. fuxinensis*; both labial cusps are larger than the lingual ones. The three lingual cusps increase in size distally.

Only isolated lower molars were detected. The complete one (Supplementary Fig. 2B1-3) is identified as a left m1 with a cusp formula of 3:2. The tooth crown is narrow mesially and wide distally with the distal labial cusp extending distally. The shape of the cusps suggests that these cusps were worn on their lingual and labial surfaces with the mesial one bearing the deepest wear. The lingual two cusps are subequal; each has a steep and flat labial surface and a convex lingual surface. The other two teeth (Supplementary Fig. 2C-D) are incomplete; one has the most mesial part preserved (with a flat mesial end) and the other has most of the distal part (with rounded end) preserved. These teeth appear to have few cusps and are shorter than the left m1 so that they were tentatively identified as m2s. Given these identifications, the m2 cusp formula should be 2:2.

**Hyoids:** The hyoid morphology varies in extant mammals and the hyoid apparatus has been rarely preserved and thus poorly studied in fossils (see [36] for a brief review). The hyoids of Mesozoic mammals remain as one of the areas to be explored in mammalian evolution and some progress has been made recently [1,37]. For this reason, we present the morphology of the hyoids in this study, which we think represent the most informative evidence of the multituberculate hyoids to date.

Hyoid elements have been reported from a handful Mesozoic mammals [1,6,9,37-39], but these fossils were either incomplete or known primarily in two-dimensional view. Here we report the three-dimensional morphology of the nearly complete set of the hyoid apparatus in the multituberculate *Sinobaatar pani*. The basihyal, pair of the ceratohyal, pair of the stylohyal, and one thyrohyal were recovered using CT-scanning. These elements were preserved in the basicranial region; they were displaced and closely associated with the auditory bones (Fig. 1; Supplementary Fig. 1 and 3).

The basihyal is identified as such because of its curved and nearly symmetrical morphology. Its two ends are wider than the central part of the bone. As orientated in Supplementary Fig. 3, the basihyal is dorsoventrally shallower than its anteroposterior dimension and its convex side extends posteriorly. The thyrohyal is asymmetrical; it shows a bending at its mid-point, marked by a knot on the ventral side of the bone. The distal portion is wider than the proximal portion and the distal end is the widest area of the bone. As orientated in Supplementary Fig. 3, it is transversely narrower than its dorsoventral depth. This morphology is in general similar to other fossil mammals [1, 37]. The ceratohyal is the shortest element of the hyoid apparatus; it has a curved body with distal end (which articulates with the basihyal) being wider than the proximal end (which articulates with the stylohyal). As in extant mammals [40-43], the stylohyal is a slender and long element; it is the longest element of the hyoids of *Sinobaatar pani*. The right one is preserved in close association with the stapes, although both elements were displaced. The proximal end (toward the basicranium) is wide and the distal end is narrow. Although relatively long, the stylohyal is proportionally not so long as that of *Chulsanbaatar* in which the stylohyal is about one third of the skull length [44] but is comparable to that of *Kryptobaatar* [6,38]. The stylohyal of *Sinobaatar* sp. [1] is ≥2.7 mm, much shorter than the 3.6 mm long stylohyal of *S. pani* (Supplementary Fig. 3). The epihyal was not preserved and we assume this element was not present in *S. pani*. If the assumption is correct, the possibility that the epihyal was fused either with the ceratohyal (less likely) or the stylohyal (more likely) cannot be ruled out. The reconstructed hyoid apparatus is shown in Supplementary Fig. 3.

The seven rod-like hyoid elements and the long stylohyal are similar to those of eutherians but differ from that of monotremes and marsupials [1,37,40]. It also differs from that of *Sinobaatar* sp. reconstructed by Zhou et al. [1] in which there are nine elements, including the epihyal. The reconstruction of the latter should be viewed with caution because it was based on impressions of a poorly preserved specimen. The “cerato- and epi-hyals” originally identified [1] could be reinterpreted as the stylohyal because the combination of the fragments is similar to the stylohyal reported here. Similar to the auditory bones, the similarities of the hyoids to those of eutherians are probably due to parallel evolution.

**Auditory bones:**

**Extant mammals -** The ear ossicles of extant mammals are represented by those of *Tachyglossus*, *Didelphis* and *Erinaceus*. These elements in monotremes and therians have been known for a long time [45,46], and it has been postulated that they evolved independently in the two phyletic lines [46,47], which has been supported by many phylogenetic analyses [1-3,9,48]. The dual origins of the middle ear in monotremes and therians were originally based on the morphological differences. For instance, the incus of monotremes was considered as independently transformed into a small plate medial (dorsal) to the malleus and this position must be regarded as an autapomorphic feature of monotremes [7,8]. In earlier studies, the ear of the monotremes was regarded as not a typically mammalian one so that their auditory bones were treated differently [46,47]. Although they differ considerably in morphology, the composition of the homologous elements (the stapes, incus, malleus, and ectotympanic) retains in these taxa; thus, the independent origin implies only to the way the ear bones were detached from the dentary[9].

Perhaps because of their small sizes, the detailed morphology and comparison between the two middle ear patterns have not been clear. In light of the Mesozoic mammal fossils discovered in recent years, there is a need to revisit the two patterns so that the evolutionary changes of the mammalian middle ear can be better understood. Here, we provide the high-resolution images of the auditory bones in *Tachyglossus*, *Didelphis* and *Erinaceus* derived from CT-scan (Supplementary Fig. 4). These ear bones are in original anatomical articulation. A detailed description of each auditory bone is beyond the scope of this study; instead, we briefly contrast the similarities and differences of the auditory bones of the three species as representatives of monotremes, marsupials, and placentals, respectively. The comparison serves as references as we interpret the auditory bones in extinct Mesozoic mammals.

In human anatomy [49], only the stapes, incus, and malleus are considered as middle ear ossicles and the ectotympanic (tympanic) ring has been fused to the petrosal and forms part of the external ear. However, in Mesozoic mammals and some extant mammals, such as monotremes and *Didelphis*, the ectotympanic is more or less part of the middle ear, similar to that of fossil mammals. The ectotympanic has been commonly discussed within the context of the middle ear evolution [50], as we did in this report. This also concerns the surangular. Thus, we use auditory bones to refer to these small bones.

The auditory bones of monotremes and therian are similar in their composition and general morphology; both consist of four bones: the stapes, incus, malleus, and ectotympanic, similarly orientated from the medial to the lateral (or dorsal to ventral in adult monotremes). The surangular has been lost at least in adult extant mammals. Of these bones, the body of the malleus and the incus are derived from the first pharyngeal arch and are homologous to the articular and quadrate, respectively of non-mammalian synapsids. The anterior process of the malleus (dermal gonial) is homologous to the prearticular [50,51]. The body of the malleus can be divided into a manubrial base, manubrial neck, and manubrium. The manubrium has a flat lateral (ventral) surface and spatulate tip for contact with the tympanic membrane. In these forms, the ectotympanic, homologous to the angular bone, is fully formed in a horseshoe or ring shape but lacks the anterior limb. The tympanic membrane is suspended by this bony frame.

The auditory bones of monotremes and therians are different in many aspects, as outlined below:

1. The ectotympanic of *Tachyglossus* is transversely (dorsoventrally) narrow (Supplementary Fig. 4). The malleus of *Didelphis* is transversely expanded and that of *Erinaceus* is even more so to form part of the external auditory meatus.
2. The malleus head of *Didelphis* or *Erinaceus* inflates to a globular shape, but there is no such a head in the malleus of *Tachyglossus.*
3. The malleus head bears the inferior and superior articular facets for the incus and articular facet is saddle-shaped in *Didelphis* or *Erinaceus*, but there is no such facet in *Tachyglossus.*
4. It was known that in early development of the opossum the caudal end of the gonial was perforated by a foramen for the chorda tympani nerve [52] and in monotremes the foramen is present in the embryonic and adult stages of the gonial in platypus [8]. Our CT-image confirms the identification of the foramen for the chorda tympani nerve in adult *Didelphis* recognized by Wible and Spaulding [13]. A groove and a short foramen on the anterior process were tentatively identified as for the chorda tympani in *Tachyglossus.*
5. The anterior process of the malleus is straight and in parallel contact with the dorsal limb of the ectotympanic, similar to the fossil taxa (Supplementary Fig. 9). In *Didelphis* or *Erinaceus*, the anterior process of the malleus is curved and wrap around the dorsal limb of the ectotympanic.
6. The orbicular apophysis is present on the ventral end of the malleus base in *Erinaceus*, although not so inflated. Presence of the orbicular apophysis characterizes the microtype of middle ear [46] that differs from the “ancestral type” represented by *Didelphis* that lacks the orbicular apophysis [53]. There is no orbicular apophysis in *Tachyglossus.*
7. The incus of *Tachyglossus* is thin, platelet-like, and slightly concave dorsally; its long and short processes are also thin and are not well delimited from the body. The incus of *Didelphis* or *Erinaceus* has an inflated body, a long process that ends with the lenticular process, and a blunt and conical short process. The lenticular process bends nearly 90° from the long process.
8. The platelet incus of *Tachyglossus* is dorsal to the body of the malleus and the contact between the two bones is flat. In *Didelphis* and *Erinaceus* the incus is caudal to the malleus and the articulation is saddle-shape.
9. The stapes of *Tachyglossus* is columnar and T-shape in lateral view, lacking the process for the stapedius muscle, whereas the stapes of *Didelphis* has two crura, a small stapedial foramen, and a small process for the stapedius muscle on the posterior crus near the head. The stapes of *Erinaceus* is stirrup-shaped with a large stapedial foramen.
10. The stapedial footplate of *Tachyglossus* is nearly circular, whereas that of *Didelphis* or *Erinaceus* is oval.
11. The stapedial footplate of *Tachyglossus* is parallel to the orientations of the incus, the malleus-incus contact, and the plane of the malleus and ectotympanic. In *Didelphis* and *Erinaceus*, the stapedial footplate is nearly parallel to the plane formed by the malleus and ectotympanic, but is perpendicular to the malleus-incus articulation.

**Ectotympanic of *S. pani* -** The ectotympanic is sickle-shaped and consists of a dorsal limb and a ventral limb (Fig. 2; Supplementary Fig. 6). Although the ventral limb is more extensively developed than in the eutriconodontan *Liaoconodon* and stem therian *Origolestes* (Fig. 3; Supplementary Fig. 9), the tympanic notch (incisure), the gap between the ends of the two limbs, is still wide. The dorsal limb is relatively straight and extends posteriorly in opposite direction to the anterior process of the malleus. The ventral limb is a partial circle and is on the anteroventral side of the bone, with its free tip curving ventroposteriorly. The limbs that bear the tympanic sulcus (sulcus tympanicus) can be seen in the medial view; thus, the orientation or the left or right ectotympanic can be identified based on these features (Supplementary Figs. 5 and 6).

The dorsal limb is plate-like, reminiscent of the plate-like ectotympanic of *Arboroharamiya* [36,54]. On its lateral surface there is an extensive contact area that is broad and shallow posteriorly but narrow and deep anteriorly; this contact area is for the anterior processes of the malleus and surangular; this contact relationship (Fig. 2; Supplementary Fig. 5) is similar to that of monotremes [8] in which the anterior process of the malleus overlaps the ventral (lateral) side of the dorsal limb of the ectotympanic. The conjunction of the posterior and ventral limbs is the thickest region of the ectotympanic and shows an angulation, but the anterior limb is absent as in extant mammals in which the auditory bones are fully suspended in the basicranial region. In anterior and lateral views, the ventral limb gradually narrows toward its tip. In lateral or medial view, the ventral limb is crescent; its inner edge continues to the anterior part of the dorsal limb, forming a nearly perfect semi-circular arc. The external surface of the ventral limb is smooth.

On the medial side of the ectotympanic, the edge of the bone inflects to form the crista tympanica [13], which is weakly developed on the dorsal limb, contrasting the distinct one on the ventral limb. The tympanic sulcus extends in a curvature through the entire bone; it is shallow on the dorsal limb but deep in the ventral limb. Ventral to the sulcus on the dorsal limb, is a flat surface, identified as the recessus meatus. By its smoothness and position to the shallow sulcus, this area may have been in contact with the tympanic membrane in life. The deepest area of the tympanic sulcus is at the junction of the posterior and ventral limbs. In the middle ear of extant mammals, the sulcus accommodates the annulus fibrosus, which is the thickened circumferential rim of the pars tensa of the tympanic membrane [55]; thus, the tympanic membrane is attached to the tympanic sulcus [49]. The outline of the ectotympanic, along with the malleus, approximates the size and shape of the tympanic membrane (Fig. 3).

**Malleus and surangular of *S. pani* -** Identifications of the two mallei as the left or right one can be confidently done because of several reasons. First, each malleus so identified fits comfortably with the ectotympanic (Fig. 2; Supplementary Fig. 5). Second, the articular facet for the incus is on the posterodorsal side of the malleus body and the facet is bounded by a narrow medial lip; this configuration makes sense physically as the sound waves reaching the tympanic membrane from the lateral side and the malleus could better mobilize the incus (Fig. 3). A similar configuration is present in the malleus of *Liaoconodon* [9] and *Origolestes* (Supplementary Fig. 9)[3]. Moreover, the foramen for the chorta tympanic on the medial side of the malleus is highly similar to those in *Lambdopsalis* [5] and *Didelphis* (Supplementary Fig. 4)[13]. Although noted in the multituberculate *Jeholbaatar* [2], the element identified as the malleus probably consists of the malleus proper, which itself is a compound bone (articular and prearticular [gonial]), and a surangular part, the bodies of the two bones are fused according to our CT data, and their anterior processes can be separated (Fig. 2; Supplementary Fig. 7). Because of this, the combined unit is more robust than the malleus of extant mammals.

CT scans revealed the suture between the anterior processes of the two bones, from which presence of a separate surangular can be deduced. The anterior process of the surangular inserts between the ectotympanic and the malleus, similar to that of *Origolestes*, and gradually tapers anteriorly to terminate as a sharp tip, resembling the surangular bone of *Arboroharamiya allinhopsoni* [54]. The suture extends for half the length of the unit and then becomes invisible, which suggests fusion of the surangular and malleus bodies. CT-scan did not detect any suture in the fused bodies of the surangular and malleus, similar to that of *Morganucodon*, so that their separation is somewhat arbitrary. The surangular part is posterodorsal to the malleus. Its posterior part is thick and medially convex with a smooth surface. The surangular boss slightly bends laterally; its position is similar to that of the malleus of *Liaoconodon* [9] and *Origolestes* (Supplementary Fig. 9)[3]. The posterior end of the surangular contributes to the dorsal end of the articular facet for the incus, also similar to *Liaoconodon* and *Origolestes*. This feature resembles those of non-mammalian cynodonts, such as *Cynognathus* and *Morganucodon*. In *Cynognathus* the surface for the articulation of the quadrate is formed by the surangular and the articular, whereas in *Morganucodon* the surangular forms a considerable part of the articular surface [56]. Based on comparison of the relative position and size of the surangular and malleus in *Morganucodon*, *Liaoconodon*, and *Origolestes* and the trajectory of the suture present in *Sinobaatar* we reconstructed a hypothetical separation between the bodies of surangular and malleus (See Fig. 3 dashed line). Arguments used to support identification of the surangular and its division from the malleus were summarized in the comparison of the surangular (see below).

The malleus has a thick “head”, a transversal part [8] or osseous lamina [13], a neck, a base for the manubrium, the manubrium, and an anterior process that is presumably homologous to the prearticular in non-mammalian cynodonts. On the lateral side, the boundary between the malleus and surangular is interpreted as the groove that leads to the foramen for the chorda tympani nerve.

The groove and foramen for the chorda tympani nerve are clear on the anterior process of the malleus, similar in position and shape to those of *Lambdopsalis* [5], *Sinobaatar* sp. [1] and *Didelphis* (Supplementary Fig. 4)[13], but differ in position and size from that in *Jeholbaatar* as interpreted originally [2]. The foramen pierces the anterior process, passes through a canal, and exits between the anterior processes of the surangular and malleus; the canal does not penetrate what has been identified as the anterior process of the surangular.

The posterior part of the fused malleus-surangular unit is thick as if it was a “head”. On the medial side, this area is smoothly convex, similar to that of *Lambdopsalis* [5]. But the “head” differs from that of therians in which the head has a spherical shape (Supplementary Fig. 4) and bears the articular facet on its caudal side. Nonetheless, such a thick area accounts for a significant mass of the bone; thus, it may function for balancing or imbalancing the mass center that could affect the frequency the ossicular system transmits [46]. At the posterior end of the “head”, a crescent and smoothly concave notch is the articular facet for the incus. The facet is primarily within the malleus, and as mentioned above, the surangular contributes to its dorsal part. The facet is laterally braced by a narrow band of bone (malleus), which we call the lateral lip; thus, the articular facet is located on the posteromedial end of the malleus-surangular unit, a condition similar to that in *Liaoconodon* and *Origolestes* (Supplementary Fig. 9). The lateral lip braces the articular facet so that sound waves reach to the lateral side of the tympanic membrane can be efficiently transmitted from the malleus-ectotympanic to the incus, stapes, and then the inner ear (Fig. 3).

The transversal part is transversely thinner than the “head”. Between the articular facet and the manubrium base, the transversal part shows an elongate neck; the posterior border of the neck is shallowly concave; thus, the posterior border of the malleus shows a “double concavities” with the ventral one being shallower than the dorsal one. This morphology is somewhat similar to that of therians and monotremes (Supplementary Fig. 4), although differences exist owing to the shift of the articulation in therians and monotremes.

Ventral to the neck, the malleus thickens both laterally and medially to form the base of the manubrium. The manubrial base is not a well-defined structure; it is “applied to the area of confluence of neck, manubrium, lateral process, orbicular apophysis and the lower part of the anterior process” [12]. A step-like border is formed between the thick base and the neck and manubrium (Supplementary Fig. 7). A small projection at the lateral side of the manubrial base is identified as the lateral process, whereas a smaller one on the medial side is the muscular process for the tensor tympani muscle; the presence of the latter evidences the tensor tympanic muscle insertion on the malleus, similar to that in extant mammals [12,13]. An orbicular apophysis was not developed; if present it would be a bulky mass at the ventral end of the manubrial base [12]. A step-like configuration is between the base on one hand and the manubrium and neck on the other. Because of this distinct border, the manubrial base in *Jeholbaatar* has been mis-identified as the incus [2], as we mentioned above. In the light of the new fossil reported here, we think the incus of *Jeholbaatar* yet to be found.

The manubrium is a thin prong projecting anteriorly and tapers towards its tip, similar to that of *Jeholbaatar*. In life, the manubrium presumably inserted in the tympanic membrane. In relation to the reconstructed size of the tympanic membrane, as inferred from the ectotympanic and malleus, the manubrium is proportionally shorter than that in extant mammals (Supplementary Fig. 4).

The anterior process of the malleus is completely fused to the transversal part and overlaps with the contact region of the ectotympanic (Supplementary Fig. 7), similar to those in other multituberculates [1,2]{Chang, 2009 #858}, monotremes [8], and some therians [13,45]. The foramen for the chorda tympani is distinct, as mentioned above. Again, the presence of the foramen echoes the view that the anterior process is homologous to the prearticular in non-mammalian cynodonts [8,15,17,52]. Although the boundary between the malleus body and the anterior process (gonial = prearticular) is not present, the position of the foramen, which should be in the anterior process, may serve as a reference to show the rough size of the anterior process.

**Incus of *S. pani* -** The best interpreted articular relationship with the malleus shows that the incus (the only one preserved) is from the right side (Supplementary Fig. 5). The incus is quite flat but is different from the platelet-like incus in monotremes (Supplementary Fig. 4). The incus has a body that has a convex side, which we interpret as the articular surface for the malleus; thus, this is the lateral side of the incus. The medial side of the bone is relatively flat with an uneven surface. Differing from that of monotremes, a long (stapedial) process exists; it has a bending angle from the body and a ridge between the body and process is present (Supplementary Fig. 8). The distal end of the long process flares to give the shape of a lenticular process. However, unlike the incus in therians, there is no bending of the purported lenticular process to the long process. The flat distal end of the lenticular process is most likely the articular facet for the head of the stapes, although this identification is uncertain because of the displaced auditory bones. A short process is not distinct, which may have been broken. If the articulation reconstructed in Supplementary Fig. 5 is correct, then, the broken short process should be at the position reconstructed in Supplementary Fig. 8. The position of the incus relative to the malleus is similar to those of *Liaoconodon* and *Origolestes*, but different from that of *Yanoconodon* [57] in which the ectotympanic, malleus and incus were interpreted as nearly the same as in the adult *Ornithorhynchus*, with a platelet-like incus abuts the medial (dorsal) side of the malleus.

**Stapes of *S. pani* -** The stapes has its footplate more or less completely preserved and may have a small corner folded (Supplementary Fig. 8). It is oval in medial (proximal) view and convex medially toward the fenestra vestibuli; its lateral surface is concave. The general shape of the footplate is similar to that of the Jurassic multituberculate *Pseudobolodon* [58] and the stem therian *Origolestes* [3]. The crura were crashed and difficult to reconstruct and interpret. From the preserved segments, it is most likely that there are two crura, the anterior one positions close to the center of the footplate, as in *Origolestes*, and the posterior on is at the edge of the plate, similar to that of *Pseudobolodon*; thus, there should be a sizable stapedial foramen. One segment is thicker than others and bears an expanded “head” which may represent the head of the stapes; by position, it unlikely represents the process for insertion of the stapedial muscle [59], as in *Arboroharamiya* [54]. However, a meaningful reconstruction of the stapes is difficult.

**Comparison:** In allotherians, auditory bones were reported from *Lambdopsalis* [5,60,61], *Chulsanbaatar* [62], *Kryptbaatar* [6,38], *Arboroharamiya* [36,54,63], *Vilevolodon* [39], *Sinobaatar* sp. [1], *Pseudobolodon* [58], and *Jeholbaatar* [2]. Most auditory bones in the reported forms are fragmentary with the exception of *Arboroharamiya allinhopsoni* and *Jeholbaatar*, in which the auditory bones were claimed to be complete. However, the specimens of these two taxa are flattened and key features such as the incudomalleolar articulation are not exposed. Due to the preservation some structures have been misidentified in *Jeholbaatar*, as discussed above. In addition, three dimensionally preserved auditory bones are known from the Early Cretaceous eutriconodontan *Liaoconodon* and the symmetrodontan *Oligolestes* [3,9] (Supplementary Movies 5 and 6). These taxa provided by far the best known morphologies for us to understand the ancestral phenotypes of mammalian middle ear. Our comparison will focus on these forms (Supplementary Fig. 9) as well as representatives of extant mammals (*Tachyglossus*, *Didelphis*, and *Erinaceus*; Supplementary Fig. 4). By these ancestral phenotypes, we may reconstruct the evolutionary transition from the quadroarticular jaw articulation in non-mammalian cynodonts to the incudomalleolar joint in monotremes and therians and test whether the evolutionary changes and development, or phylogeny and ontogeny, of the middle ear in mammals are related.

**Ectotympanic -** In general shape, the ectotympanic shows a longer and more anteromedially extended ventral limb than those in *Liaoconodon* and *Origolestes*. The outline of the ectotympanic in *Sinobaatar* sp. [1], based on impression in the matrix, is generally similar to that of *Jehobaatar* [2]. The plate-like lateral limb is uncommon in mammals but reminiscent of the plate-like ectotympanic of *Arboroharamiya* [36,54]. The ectotympanic bone is similar to those of extant adult mammals in completely lacking the anterior limb [8,13,45,47]. This is one of the characters for the definitive mammalian middle ear that is fully separated from the dentary [51].

In early embryonic stage of marsupials, the ectotympanic is a three-pronged bone, including the anterior limb [14,15,18,19,64], similar to that of *Liaoconodon* (Supplementary Fig. 9)[9]. During the ontogeny, the anterior limb is resorbed while the dorsal and ventral limbs gradually elongated to form a more complete horseshoe or ring-shaped frame in which the tympanic notch is small. It is notable that during this process, the dorsal limb is relatively straight, whereas the ventral limb is more curved. In the stem therian *Origolestes*, the anterior process of the ectotympanic was greatly reduced but still existed [3]. The ventral limb of the ectotympanic in *S. pani*, presumably homologous to the reflected lamina of the angular, is more extensively developed and curved backward than those of *Liaoconodon* and *Origolestes*. Thus, the bony frame primarily formed by the ectotympanic and the malleus provided substantial support for the tympanic membrane, more so than in *Liaoconodon* and *Origolestes*.

However, the tympanic notch (incisure) is still wide; this contract the ring-like or horseshoe-shaped ectotympanic in extant mammals (Supplementary Fig. 4)[12,45,47]. In adult individuals of extant mammals, the tympanic membrane is completely suspended by the bony frame of the ectotympanic. As in the ectotympanic of other mammals, such as the Tertiary multituberculate *Lambdopsalis* [5], Late Cretaceous multituberculate *Kryptobaatar* [6], and extant mammals [47], the inner surface of the gently curved bone is grooved, forming the tympanic sulcus, where the tympanic membrane was attached. The tympanic sulcus, although quite shallow, extends fully along the inner rim of the ectotympanic, differing from the condition in *Liaoconodon* and *Origolestes* in which the sulcus was not fully developed in the dorsal limb (Supplementary Fig. 9).

The ectotympanic of *Sinobaatar pani* is more similar to those of extant mammals than to any known Mesozoic mammaliaforms. We consider that loss of the anterior limb, more fully developed ventral limb, and fully developed tympanic sulcus of the ectotympanic derived characters compared to those of *Liaoconodon* and *Origolestes*; this is consistent with the fact that *Sinobaatar pani*, as in other multituberculates, acquired the definitive mammalian middle ear. These evolutionary changes in the Mesozoic mammals are similar to the developmental process of the ectotympanic in extant mammals.

**Malleus** - The malleus, along with the ectotympanic, reiterates the previous view that the auditory bones are fully detached from the dentary in multituberculates; it also reconfirms presence of the foramen for the chorda tympani in the anterior process, thus, the homology of the anterior process to the prearticular of non-mammalian cynodonts [5,6,38,60-62].

The malleus of *Sinobaatar* *pani* provides the unambiguous evidence about the articular facet for the incus in multituberculates. The braced hinge joint differs from the dorso-ventral relationship of *Jeholbaatar*, interpreted by Wang et al. [2] and from the antero-posterior relationship illustrated in *Sinobaatar* sp. [1]. As described above, the shape, position, and formation (primarily by the malleus, with contribution from the surangular) of the braced hinge joint are similar to those of the eutriconodontan *Lioconodon* and the stem therian *Origolestes*, although the bone was probably more vertically positioned in the latter. This implies that the malleus-incus relationship is neither rostrocaudal, as in therians, nor ventrodorsal, as in monotremes. The articulation is simpler than the saddle shaped articulation of therians but more complex than the flat abutting pattern of monotremes (Supplementary Fig. 4). Such an articulation is reminiscent of the quadrato-articular synovial joint in morganucodontids [56,65]; in the latter, however, the joint still functioned for jaw suspension.

In monotremes, an incudomalleolar synovial joint is absent, and the flat platelet incus abuts on the dorsal side of the transversal part of the malleus (Supplementary Fig. 4)[7,8]. In therians, the head that bears the articular facet is inflated into a more or less spherical shape. The articular facet on the posterior side of the head can be divided into the inferior and superior parts so that the facet is saddle-shaped and articulates the incus posteriorly [12,13,45-47]. The relative position between the malleus and incus of *Sinobaatar* *pani* is similar to that of *Liaoconodon* and *Origolestes* in which the incus is on the posteromedial side of the malleus body. This relationship is somewhat intermediate between the therian condition and the monotreme condition (Supplementary Fig. 4) and between the quadrato-articular synovial joint in morganucodontids and the saddle-shaped joint in therians.

The anterior process is similar in relative size to those in some extant mammals, such as monotremes and marsupials [7,8,13,17-19,47,66] and to *Origolestes*, but shorter than that of *Liaoconodon*. It acts as a rigid link connecting the malleus to the ectotympanic, as in extant mammals [46]. Development of the malleus neck in *Sinobaatar pani* is similar to that of extant mammals, particularly therians, which is more derived than the condition in *Liaoconodon* and *Origolestes*. In the latter, the neck of the malleus is not formed so that the general shape is more similar to the retroarticular process of basal mammaliaforms and in the posterior border of the malleus, the “double-concavities” outline is absent. The neck was reported in *Liaoconodon* [9], but in the light of the malleus of *S. pani*, that identification is incorrect. Similarly, the neck is not developed in the stem therian *Origolestes* (Supplementary Fig. 9). Development of the neck increases the lever ratio of the ossicular chain for sound transmit [46] so that it would increase the efficiency to amplify sound pressure acting at the stapedial footplate.

The manubrium of the malleus in *Sinobaatar pani* and *Jeholbaatar* is unequivocal, compared to other Mesozoic mammaliaforms, including *Liaoconodon* and *Origolestes*. The complete retroarticular process was unknown in *Morganucodon* [56,67], although the manubrium was thought to be equivalent to the retroarticular process [14,50,56,68]. The manubrium has now been considered as a neomorphic outgrowth from the base and the retroarticular process may be equivalent to the neck of the malleus and the manubrial base [10,67]. This view gains support from developmental studies [66,69-72]. In Mesozoic mammals, the eutherian *Uchkudukodon nessovi* has the manubrium preserved [73,74]; it is derived from an inflated base, similar to the condition in *S. pani* and *Jeholbaatar*. In extant mammals the manubrium is a long thin process usually with a spatulate tip [75]; it inserts in the three-layered tympanic membrane [70] and commonly reaches to the center of the membrane.

The manubrium in multituberculates (*S. pani* and *Jeholbaatar*) is distinctively thinner than the base. In *Liaoconodon* the manubrium was interpreted as absent [9], whereas in *Origolestes* it was considered to be present. However, in *Origolestes* the manubrium base is not inflated and the purported manubrium is gradually continuous with the base, differing from that of *S. pani*. In the light of the new specimen of *S. pani*, we would argue that a true manubrium has not yet developed in *Origolestes* (Supplementary Fig. 9). The ventral process of the malleus in *Liaoconodon* and *Origolestes* is better interpreted as homologous to the retroarticular process, or part of it. The manubrium of *S. pani* is shorter than that of extant mammals and does not have a spatulate distal end. However, because the tip of the manubrium is procartilaginous and blastematous [52], it may not be preserved in fossils. Given the current working hypotheses, the manubrium as a neomorphic feature must have evolved independently in multituberculates, monotremes and therians; it was probably developed along with formation of the manubrial neck; both structures would increase the lever ratio for sound transmit of the ossicular chain [46,76]. Comparing the malleus morphologies within the material available (*Liaoconodon*, *Origolestes*, *Sinobaatar* *pani*; Supplementary Fig. 9), we concur with the hypothesis that the manubrium base and neck are probably equivalent to the retroarticular process (see below), whereas the manubrium is a neomorphic outgrowth [51,71].

Another feature related to the manubrium is the orbicular apophysis, which characterizes the microtype middle ear of therians that differs from the ancestral therian ear type; the latter is typically represented by *Didelphis* (Supplementary Fig. 4)[46,53,77]. As a considerable imbalancing or balancing mass of the malleus [46], the orbicular apophysis increases ossicular inertia about the anatomical axis of rotation, but its adaptive function in a high-frequency ear is still not clear [53], although other studies show it is dispensable for normal hearing and balance in mammals [78]. The orbicular apophysis is another structure relevant to the discussion of the homology and evolution of the malleus. In contrast to the neomorphic manubrium, the orbicular apophysis arises from cranial neural crest cells within the second pharyngeal arch, similar to the retroarticular process; thus, it has been considered homologous to the retroarticular process [71]. In this regard, however, some confusion in terminology is current. As pointed out by Mason [53,79], in the developmental biology literature, the orbicular apophysis is commonly referred to as the “processus brevis” [71,78,80], but the term “processus brevis” originally referred to the lateral process of the malleus, i.e. the projection of the proximal manubrium where it inserts into the peripheral part of the tympanic membrane pars tensa [12,45,81]. Henson [75] specified that “The orbicular apophysis is a bony projection which extends posteriorly from the manubrial base; it may be large and massive, or small and scarcely recognizable….Projecting into the tympanic membrane from the manubrial base is the lateral process (processus lateralis, brevis). This process is usually small and indistinguishable in gross specimens, but is seemingly consistent in occurrence and is always capped by, or composed entirely of, cartilage.” Thus, the orbicular apophysis and the true processus brevis are separate structures. Given that its absence in the ancestral therian ear type, such as *Didelphis*, and in any Mesozoic mammals (Supplementary Fig. 9), the orbicular apophysis must be a derived feature within therians. From an evolutionary perspective, the structure homologous to the retroarticular process should be present in species that lacks the orbicular apophysis, such as *Didelphis*; similarly, the same assumption can be made for the Mesozoic forms (Supplementary Fig. 9). If the retroarticular process may be equivalent to the neck of the malleus and the manubrial base [51,67], then the orbicular apophysis has to be only a ventral extension of the neck and/or the base of the malleus, an assumption that needs further test from developmental studies.

We propose that the braced hinge joint in eutriconodontans, stem therians, and multituberculates represent a derived condition compared to the primary quadroarticular joint in non-mammalian cynodonts, such as morganucodontids, but it retains the general convex-concave relationship of the quadrato-articular articulation, as recognized previously [46]. This braced hinge joint is unquestionably a primitive condition compared to the monotreme and therian conditions (Supplementary Fig. 4) so that it can be regarded as an ancestral for mammals. This or similar pattern may have given rise to those of monotremes and therians. We also propose that the *Sinobaatar* condition is more derived than those of *Liaoconodon* and *Origolestes* in having the malleus neck and true manubrium.

We consider presence of the anterior process, subequal to the length of the posterior process of the ectotympanic, as a primitive mammalian condition, and in advanced forms it is greatly reduced. Presence of the inflated malleus head bearing the articular facets on its posterior side is a derived therian condition. The manubrial neck and the manubrium are derived conditions developed within mammals independently in monotremes, therians, and multituberculates. Although the neck may be homologous with the articular or the retroarticular process, the manubrium is a neomorph that probably evolved independently. The orbicular apophysis is a derived condition in therian mammals. Although the status of the surangular remains unclear (see below), the evolutionary changes of the malleus observed in fossils are comparable to its developmental morphogenesis in extant mammals [5,20,25-27]. In particular, the malleus base and manubrium develop and ossify later than other parts of the malleus.

**Surangular -** In the original study, only the surangular boss, not the surangular, was identified in *Liaoconodon* [9]. With the high-resolution CT-scan images available now, it is confirmed here that a separate surangular is present in the auditory bones of *Liaoconodon* (Supplementary Fig. 9; Supplementary Movie S5). Thus, the surangular or surangular part has been reported in the euharamiyidan *Arboroharamiya* [54] and *Vilevolodon* [39], the eutriconodontan *Liaoconodon*, the symmetrodontan *Origolestes* [3] and the multituberculate *Jeholbaatar* [2] and *Sinobaatar pani* (this study). The identification of the surangular in Mesozoic mammals becomes increasingly convincing not only because it has been identified in several groups but also because in *Liaoconodon*, *Origolestes*, *Arboroharamiya*, and multituberculates the position of the surangular is highly similar in relation to the malleus. In all these forms, the surangular is located dorsolateral to the malleus and its posterior part contributes to the formation of the articular facet for the incus. Kermack et al. [56] stated: “In *Cynognathus*, this bone [surangular] shares with the articular in the formation of the actual bearing surface of the jaw-joint---about a third being formed by the surangular; …In *Morganucodon*, the surangular forms a considerable part of the articular surface.” The surangular identified in the aforementioned taxa shows the continuity with their close kin and that it did not disappear abruptly during the transition from postdentary unit in the non-mammalian cynodonts to mammals.

Here we summarize the arguments for identification of the surangular and the reasons to arbitrarily draw the line separating it from the malleus at the fused malleus-surangular body in *Sinobaatar pani*: 1) A small surangular or surangular part has been identified in several taxa, including *Liaoconodon*, *Origolestes*, *Arboroharamiya*, *Vilevolodon*, and *Jeholbaatar*. Presence of the surangular in these forms makes sense in an evolutionary perspective, consistent with the fact that the surangular was the major element holding the postdentary unit to the dentary before the attachment. 2) The position of the surangular dorsolateral to the malleus is consistent in all of the forms. 3) The surangular contributes to a small part on the dorsolateral side of the articular facet for the incus in *Liaoconodon* and *Origolestes*, consistent with our identification in *S. pani*; such a configuration is also consistent with the articular-incus articulation of *Morganucodon* in which the surangular made a contribution. 4) The foramen for the chorda tympani nerve is clearly present. In extant mammals, such as *Didelphis* (Fig. S4E-H), this foramen penetrates the anterior process of the malleus (prearticular) so that on the lateral and medial sides of the process, the opening is clear. However, in *S. pani*, the foramen did not penetrate the anterior process of the surangular but exits at the suture between the two processes. 5) The suture between the anterior processes of the surangular and malleus is distinct in CT scans (see Fig. S7). 6) The malleus-surangular unit is robust (both the body and the anterior process[es]), compared to the malleus of extant mammals, which is again consistent with the evolutionary trend that the surangular was gradually reduced instead of suddenly disappeared. In extant mammals, the malleus is very thin except for the inflated head that bears the articular facet for the incus in therians, whereas the fate of the surangular is uncertain (see below). 7) The general morphology of the auditory bones of *S. pani* is more derived than those of *Liaoconodon* and *Origolestes*. The fusion of the malleus and surangular is consistent with the general pattern. Within the evolutionary trend, it is also sound that the surangular part should not be proportionally large in relation to the malleus in *S. pani*.

Reduction of the surangular must be a major step toward the separation of the postdentary elements from the dentary; it also frees more area on the medial surface of the dentary for attachment of jaw muscles (see [9] for a review). In extant mammals, the surangular has been considered to be homologous to the accessory malleus (the ossiculum accessorium mallei), a small bone lying above the anterior process of the malleus in some extant mammals at embryonic stage of ontogeny [52,67,75,82]. However, the observations and interpretations appear to be inconsistent in the literatures. For instance, Watson [83] noted: “The ossiculus acessorium malleoli of a sheep and of Xenartha has a characteristic relation to the upper limb of the tympanic and to the posterior part of Meckel's cartilage. It seems, therefore, to be a bone of the reptilian lower jaw, and it is not improbable that it is really the surangular.” In his study of the development of the auditory ossicles of the opossum (*Didelphys virginiana*), McClain [52], following van Kampen [84] and Watson [83], made some observations on a small and independently developing cartilage element lateral to the malleus that eventually contributed to the lateral ridge of the malleus and postulated this element as possibly homologous to the surangular. Henson [75] wrote: “In mammals there is occasionally a small bone lying above the anterior process of the malleus; this has been called the accessory malleus (ossiculum accessorium mallei) and has been interpreted to be a remnant of the reptilian surangular [82].” Similarly, Macrini [85] stated: “Furthermore, the surangular, angular, and prearticular of the non-mammalian synapsid lower jaw are homologous to the os goniale, ectotympanic, and ossiculum accessorium mallei, respectively, of the mammalian ear [14,17,86] .” Although Shute [82] wrote that in mammals “the surangulare is probably lost or reduced to the occasional ossiculum accessorium mallei”, he also pointed out that McClain [52] “described a cartilage lateral to the malleus which he homologized, tentatively and most improbably, with the membranous surangulare. His account and figures suggest that this structure may have been the cartilage of Spence.” Wible and Spaulding [13] commented that McClain’s interpretation is unlikely in light of the position of the surangular dorsal to the prearticular in non-mammalian cynodonts, which suggests that if present in the opossum it should be near the gonial. As pointed out by Han et al. [54], although the ossiculum accessorium mallei as a vestige surangular has been noted in developmental and morphological studies [67,87,88], there seems no clear evidence that confirms the presence of the surangular and/or the nature of the accessory malleus in early ontogeny of extant mammals in more recent studies [17,66,70,87,89-91].

Maier and Ruf [88] provided the most recent treatment of the subject in cetartiodactyls, in which a bony process of considerable size in the malleus was identified as the processus internus praearticularis. In earlier literatures, this process was regarded as ‘ossiculum accessorium malleoli’ of Hagenbach [92]. Recognizing that its homology has been disputed, the authors concluded that the ‘ossiculum accessorium malleoli’ of Hagenbach [92] is only an internal process of the prearticular and hence should be renamed ‘processus internus praearticularis’.

In short, the fate of the surangular remains to be explained during the evolution of mammals [9]. Accumulating evidence from eutriconodontans, stem therians, haramiyidans, and multituberculates [2,3,9,54] has been making an increasingly compelling case that the surangular had persisted into the middle ear of Mesozoic mammals. Its absence in adult extant mammals may be a result of the continuous reduction of ossicle mass during the mammalian evolution for efficient hearing of high frequency sounds. The surangular probably remained as a trace element in early ontogeny of some extant mammals, a hypothesis calling for test from developmental biological studies [93].

**Incus** - The incus displays a morphology that differs from any known incus of mammals, including multituberculates. In most taxa, the incus was preserved incompletely [58,60,62]. In *Sinobaatar* sp. [1] the incus and other auditory bones were reconstructed from impressions so that there is little detail of the bones. In *Jeholbaatar*, the manubrium base was mis-identified as the incus; the incus remains unknown in this taxon. The incus of *Sinobaatar* *pani* represents the first incus with relatively complete morphology in known multituberculates. Similar to that of monotremes, the incus is quite flat, but differs from the latter in having a distinctive long process, a convex articular surface for the malleus, and being positioned on the posterodorsal side of the malleus, as reflected by the position of the articular facet on the malleus. Presence of a long process is similar to therians, but in contrast to the therians the incus of Sinobaatar is relatively flat, has a convex articular surface for the malleus, and a straight lenticular process.

As discussed above, the incus-malleus articulation is more similar to that of *Liaoconodon* and *Origolestes* than to any known mammals. However, the middle ear bones of another eutriconodontan, *Yanoconodon*, were considered similar to that of monotremes. As observed by Luo et al. [57]: “The outline and proportion of the ectotympanic, malleus and incus of *Yanoconodon* are similar to their homologues in adult *Ornithorhynchus* (except for the gracile tips of the manubrium and ectotympanic in the latter).” Thus, the middle ear of *Yanoconodon* was reconstructed as similar to that of *Ornithorhynchus* with the incus being platelet like and abut to the dorsal (medial) side of the malleus. A similar reconstruction was published for *Jeholbaatar* [2], although the evidence is insufficient.

**Stapes -** Evolution of the stapes has been discussed in detail in several studies [3,36,58,59,61,63,94]. Various morphotypes of the stapes (columelliform and microperforated, robust and rod-like, and bicrural) in multituberculates have been suggested based on fragmentary specimens (see [58] for a brief review). The stapes of the Jurassic multituberculate *Pseudobolodon* is bicrural and represents the earliest known multituberculate stapes [58]. The stapes of *Jeholbaatar* [2] and *Sinobaatar* sp. [1] were reconstructed as rod-like, but in both species the stapes were likely partly preserved. Although the stapes of *Sinobaatar pani* is damaged, it is more likely bicrural than column or rod-like. The shape of the footplate is generally comparable to that of *Pseudobolodon* and also similar to that of the stem therian *Origolestes* in having an oval outline and convex (toward the fenestra vestibuli) footplate.

**Euharamiyidan auditory bones –** The relationship of “haramiyids” with other mammaliaforms has remained controversial even better materials of euharamiyidans and other Mesozoic mammals were discovered in recent years [2,4,36,39,54]. There are several competing hypotheses: Multituberculates and euharamiyidans form a sister group with *Haramiyavia* and *Thomasia* as the outgroup within Mammalia; multituberculates and euharamiyidans form a sister group within Mammalia, but *Haramiyavia* and *Thomasia* are outside Mammalia, even grouped with tritylodontids; finally, “haramiyids” as a whole are outside of Mammalia but multituberculates stay as a member of Mammalia. These competing hypotheses are at least partly affected by character coding in phylogenetic analyses in which the auditory features and related ones have played a critical role. The auditory bones are known in *Arboroharamiya* [36,54,63] and *Vilevolodon* [39]. There are at least two major issues related to these euharamiyidan auditory bones: the difference of the auditory bones between *Arboroharamiya* and *Vilevolodon* and the deviation of these bones from those of other mammaliaforms, as reviewed in a recent study[36].

In short, *Vilevolodon* was interpreted as retaining a mandibular middle ear, a primitive form similar to that of morganucodontids but relatively derived in having a short anterior limb of the ectotympanic and a shortened prearticular/Meckel’s cartilage that was lodged in a short postdentary trough on the dentary. Whether it has a double jaw joint is unclear. In contrast, *Arboroharamiya* has all the auditory bones detached from the dentary, thus the definitive mammalian middle ear (DMME). Among the auditory bones of *Vilevolodon*, the ectotympanic may be interpreted as the stylohyal [36]; others, such as the incus, are fundamentally different in morphology from those of *Arboroharamiya*. For the two genera coming from the same group of euharamiyidans, possessing similar dentition, and having similar temporal and paleogeographic distributions, their differences in the auditory region appear unreal. New fossil evidence is needed to settle this down.

For the second issue, the reconstructed mandibular middle ear of *Vilevolodon*, if correct, is definitely more primitive than any known middle ear of mammals, such as that of *Liaoconodon* [9]; thus, it is beyond comparison with the DMME of *Sinobaatar pani*. Although both *Sinobaatar* and *Arboroharamiya* have the DMME, that of *Arboroharamiya* is peculiar and shows significant deviation from the middle ear commonly known in mammals. As concluded by some authors[36], even the stapes and incus, the most unambiguous auditory bones of *Arboroharamiya*, are highly different from those of other known mammaliaforms; the malleus and ectotympanic are even more unusual compared with the corresponding elements in known mammaliaforms, not to mention the unique surangular. Now, more evidence shows that presence of the surangular is probably not uncommon in Mesozoic mammaliaforms, but the morphology of the surangular of *Arboroharamiya* differs from those of others (*Liaoconodon*, *Origolestes*, and *Sinabaatar pani*). Some detailed morphologies, such as the articular facet for the incus on the malleus is unclear in *Arboroharamiya*. However, it seems true that presence of the surangular and an incomplete ectotympanic are primitive, or ancestral, auditory features for mammals. We would interpret the unique auditory bones of *Arboroharamiya* to be autapomorphy for the genus; it cannot be generalized for euharamiyidans until we have a better understanding of the middle ear of *Vilevolodon* and other new evidence. Therefore, there seems no resolution for this complex issue based on available but controversial evidence and interpretations. Nonetheless, in terms of morphology we view the difference of auditory bones between *Arboroharamiya* and *S. pani* to be more distinct than those between monotremes and therians; this implies either a distant phylogenetic relationship between the two taxa or a unique adaption of the auditory system for gliding locomotion of *Arboroharamiya.* These diverse and controversial morphologies show again the plasticity of the middle ear evolution in mammaliaforms, and the resolution again depends on the phylogeny; such a phylogeny needs to reconsider other taxa, including *Haramiyavia*, whose auditory apparatus also remains controversial.

**References**

1. Zhou C-F, Bhullar BAS and Neander AI *et al.* New Jurassic mammaliaform sheds light on early evolution of mammal-like hyoid bones. *Science* 2019; **365**: 276–9.

2. Wang H-B, Meng J and Wang Y-Q. Cretaceous fossil reveals a new pattern in mammalian middle ear evolution. *Nature* 2019; **576**: 102–5.

3. Mao F-Y, Hu Y-M and Li C-K *et al.* Integrated hearing and chewing modules decoupled in a Cretaceous stem therian mammal. *Science* 2020; **367**: 305–8.

4. Krause DW, Hoffmann S and Hu Y-M *et al.* Skeleton of a Cretaceous mammal from Madagascar reflects long-term insularity. *Nature* 2020; **581**: 421–7.

5. Meng J and Wyss AR. Monotreme affinities and low-frequency hearing suggested by multituberculate ear. *Nature* 1995; **377**: 141–4.

6. Rougier GW, Wible JR and Novacek MJ. Middle-ear ossicles of the multituberculate *Kryptobaatar* from the Mongolian Late Cretaceous: implications for mammaliamorph relationships and the evolution of the auditory apparatus. *Am Mus Novit* 1996; **3187**: 1–43.

7. Zeller U. Die Enwicklung und Morphologie des Sch.dels von *Ornithorhynchus anatinus* (Mammalia: Prototheria: Monotremata). *Abh Senckenb Naturforsch Ges* 1989; **545**: 1–188.

8. Zeller U. Ontogenetic evidence for cranial homologies in monotremes and therians, with special reference to *Ornithorhynchus*. In: Szalay FS, Novacek MJ and McKenna MJ (eds.). *Mammal Phylogeny: Mesozoic Differentiation, Multituberculates, Monotremes, Early Therians, and Marsupials*. New York: Springer-Verlag, 1993, 95–107.

9. Meng J, Wang Y-Q and Li C-K. Transitional mammalian middle ear from a new Cretaceous Jehol eutriconodont. *Nature* 2011; **472**: 181–5.

10. Aitkin LM and Johnstone BM. Middle‐ear function in a monotreme: The echidna (*Tachyglossus aculeatus*). *J Exp Zool Part A: Ecol Genet Physiol* 1972; **180**: 245–50.

11. Gates GR, Saunders JC and Bock GR *et al.* Peripheral auditory function in the platypus, *Ornithorhynchus anatinus*. *J Acoust Soc Am* 1974; **56**: 152–6.

12. Henson Jr OW. Some morphological and functional aspects of certain structures of the middle ear in bats and insectivores. *U Kansas Sci Bull* 1961; **42**: 151–255.

13. Wible JR and Spaulding M. A reexamination of the Carnivora malleus (Mammalia, Placentalia). *PloS one* 2012; **7**: e50485.

14. Goodrich ES. *Studies on the Structure and Development of Vertebrates* New York and London: Macmillan and Co., 1931, 1–837.

15. de Beer GR. *The Development of the Vertebrate Skull*. Oxford: Clarendon Press, 1937, 1–552.

16. Maier W. Der Processus angularis bei *Monodelphis domestica* (Didelphidae; Marsupialia) und seize Beziehungen zum Mittelohr: Eine ontogenetische und evolutions morphologische Untersuchung. *Jahrbu Morphol* *MikrosK Anat 1 Abt,* *Gegenbaurs Morphol Jahrb* 1987; **133**: 123–61.

17. Maier W. Phylogeny and ontogeny of mammalian middle ear structures. *Neth J Zool* 1990; **40**: 55–74.

18. Filan SL. Development of the middle ear region in *Monodelphis domestica* (Marsupialia, Didelphidae): marsupial solutions to an early birth. *J Zool* 1991; **225**: 577–88.

19. Clark CT and Smith KK. Cranial osteogenesis in *Monodelphis domestica* (Didelphidae) and *Macropus eugenii* (Macropodidae). *J Morphol* 1993; **215**: 119–49.

20. Kusuhashi N, Hu Y-M and Wang Y-Q *et al.* Two eobaatarid (Multituberculata; Mammalia) genera from the Lower Cretaceous Shahai and Fuxin formations, northeastern China. *J Vertebr Paleontol* 2009; **29**: 1264–88.

21. Hu Y-M and Wang Y-Q. *Sinobaatar* gen. nov.: first multituberculate from the Jehol Biota of Liaoning, northeast China. *Chin Sci Bull* 2002; **47**: 933–8.

22. Meng J. Mesozoic mammals of China: implications for phylogeny and early evolution of mammals. *Natl Sci Rev* 2014; **1**: 521–42.

23. Pan Y-H, Sha J-G and Zhou Z-H *et al.* The Jehol Biota: definition and distribution of exceptionally preserved relicts of a continental Early Cretaceous ecosystem. *Cretaceous Res* 2013; **44**: 30–8.

24. He H-Y, Wang X-L and Zhou Z-H *et al.* Timing of the Jiufotang Formation (Jehol Group) in Liaoning, northeastern China, and its implications. *Geophys Res Lett* 2004; **31**: L12605.

25. Chang S-C, Zhang H-C and Renne PR *et al.* High-precision 40Ar/39Ar age for the Jehol Biota. *Palaeogeogr Palaeocl* 2009; **280**: 94–104.

26. Eberth DA, Russell DA and Braman DR *et al.* The age of the dinosaur-bearing sediments at Tebch, Inner Mongolia, People's Republic of China. *Can J Earth Sci* 1993; **30**: 2101–6.

27. Zhou Z-H. Evolutionary radiation of the Jehol Biota: chronological and ecological perspectives. *Geol J* 2006; **41**: 377–93.

28. Chang M-M, Chen P-J and Wang Y-Q *et al.* *The Jehol Biota: the emergence of feathered dinosaurs, beak birds and flowering plants*. Shanghai: Academic Press, 2003, 1–208.

29. Wang X. Background for the Plant Fossils. In: Wang X (ed.) *The Dawn Angiosperms*. Berlin, Heidelberg: Springer, Cham, 2018, 334.

30. Zhou Z-H and Wang Y. Vertebrate assemblages of the Jurassic Yanliao Biota and the Early Cretaceous Jehol Biota: Comparisons and implications. *Palaeoworld* 2017; **26**: 241–52.

31. Yao X, Liao C-C and Sullivan C *et al.* A new transitional therizinosaurian theropod from the Early Cretaceous Jehol Biota of China. *Sci Rep* 2019; **9**: 1–12.

32. Zhang J-Y, Jin F and Zhou Z-H. A review of mesozoic osteoglossomorph fish *Lycoptera longicephalus*. *Vertebrata PalAsiatica* 1994; **32**: 41–59.

33. Zhou Z-H and Zhang F-C. A long-tailed, seed-eating bird from the Early Cretaceous of China. *Nature* 2002; **418**: 405–9.

34. Xu X, Zhou Z-H and Wang X-L *et al.* Four-winged dinosaurs from China. *Nature* 2003; **421**: 335–40.

35. Wang X-L, Kellner AW and Jiang S-X *et al.* New toothed flying reptile from Asia: close similarities between early Cretaceous pterosaur faunas from China and Brazil. *Naturwissenschaften* 2012; **99**: 249–57.

36. Meng J, Mao F-Y and Han G *et al.* A comparative study on auditory and hyoid bones of Jurassic euharamiyidans and contrasting evidence for mammalian middle ear evolution. *J Anat* 2020; **236**: 50–71.

37. Bi S-D, Zheng X-T and Wang X-L *et al.* An Early Cretaceous eutherian and the placental–marsupial dichotomy. *Nature* 2018; **558**: 390–5.

38. Wible JR and Rougier GW. Cranial anatomy of *Kryptobaatar dashzevegi* (Mammalia, Multituberculata), and its bearing on the evolution of mammalian characters. *B Amn Mus Nat Hist* 2000; **247**: 1–120.

39. Luo Z-X, Neander AI and Zhang Y-G *et al.* New evidence for mammaliaform ear evolution and feeding adaptation in a Jurassic ecosystem. *Nature* 2017; **548**: 326–9.

40. Hoffmeister RG and Hoffmeister DF. The hyoid in North American squirrels, Sciuridae, with remarks on associated musculatura. *An Inst* *Biol* *Ser Zool* 1991; **62**: 219–34.

41. Veselka N, McErlain DD and Holdsworth DW *et al.* A bony connection signals laryngeal echolocation in bats. *Nature* 2010; **463**: 939–42.

42. Pérez LM, Toledo N and De Iuliis G *et al.* Morphology and function of the hyoid apparatus of fossil xenarthrans (mammalia). *J Morphol* 2010; **271**: 1119–33.

43. Casali DM and Perini FA. The evolution of hyoid apparatus in Xenarthra (Mammalia: Eutheria). *Hist Biol* 2017; **29**: 777–88.

44. Kielan-Jaworowska Z and Gambaryan PP. Postcranial anatomy and habits of Asian multituberculate mammals. *Lethaia* 1994; **27**: 1–92.

45. Doran AHG. Morphology of the Mammalian Ossicula auditûs. *T Linn Soc London 2nd Ser:* *Zool* 1878; **1**: 371–497.

46. Fleischer G. *Evolutionary principles of the mammalian middle ear*. Berlin, Heidelberg, New York: Springer-Verlag, 1978, 1–70.

47. Fleischer G. Studien am Skelett des Gehörorgans der Säugetiere, einschließlich des Menschen. *Säugetierkd Mitt* 1973; **21**: 131–239

48. Rich TH, Hopson JA and Gill PG *et al.* The mandible and dentition of the Early Cretaceous monotreme *Teinolophos trusleri*. *Alcheringa* 2016; **40**: 475–501.

49. Standring S. *Gray’s anatomy. The anatomical basis of clinical practice, Forty-First Edition*. New York: Elsevier Limited, 2016, 1–1584

50. Allin EF. Evolution of the mammalian middle ear. *J Morphol* 1975; **147**: 403–37.

51. Allin EF and Hopson JA. Evolution of the auditory system in Synapsida (“mammal-like reptiles” and primitive mammals) as seen in the fossil record. In: Webster DB, Popper AN and Fay RR (eds.). *The evolutionary Biology of Hearing*. New York, NY: Springer, 1992, 587–614.

52. McClain JA. The development of the auditory ossicles of the opossum (*Didelphys virginiana*). *J Morphol* 1939; **64**: 211–65.

53. Mason MJ. Of mice, moles and guinea pigs: functional morphology of the middle ear in living mammals. *Hearing Res* 2013; **301**: 4–18.

54. Han G, Mao F-Y and Bi S-D *et al.* A Jurassic gliding euharamiyidan mammal with an ear of five auditory bones. *Nature* 2017; **551**: 451–6.

55. Henson MM, Madden VJ and Rask-Andersen H *et al.* Smooth muscle in the annulus fibrosus of the tympanic membrane in bats, rodents, insectivores, and humans. *Hearing Res* 2005; **200**: 29–37.

56. Kermack KA, Mussett F and Rigney HW. The lower jaw of *Morganucodon.* *Zool J Linn Soc* 1973; **53**: 87–175.

57. Luo Z-X, Chen P-J and Li G *et al.* A new eutriconodont mammal and evolutionary development in early mammals. *Nature* 2007; **446**: 288–93.

58. Schultz JA, Ruf I and Martin T. Oldest known multituberculate stapes suggests an asymmetric bicrural pattern as ancestral for Multituberculata. *P Roy Soc B: Biol Sci* 2018; **285**: 20172779.

59. Meng J and Hou S-L. Earliest known mammalian stapes from an early cretaceous eutriconodontan mammal and implications for evolution of mammalian middle ear. *Palaeontol* *Pol* 2016; **67**: 181–96.

60. Desui M and Lillegraven J. Discovery of 3-ear ossicles in a multituberculate mammal. *Natl Geogr Res* 1986; **2**: 500–7.

61. Meng J. The stapes of *Lambdopsalis bulla* (Multituberculata) and transformational analyses on some stapedial features in Mammaliaformes. *J Vertebr Paleontol* 1992; **12**: 459–71.

62. Hurum JH, Presley R and Kielan-Jaworowska Z. The middle ear in multituberculate mammals. *Acta Palaeontol Pol* 1996; **41**: 253–75.

63. Meng J, Bi S-D and Zheng X-T *et al.* Ear ossicle morphology of the Jurassic euharamiyidan *Arboroharamiya* and evolution of mammalian middle ear. *J Morphol* 2018; **279**: 441–57.

64. Ramírez-Chaves HE, Wroe SW and Selwood L *et al.* Mammalian development does not recapitulate suspected key transformations in the evolutionary detachment of the mammalian middle ear. *P Roy Soc B: Biol Sci* 2016; **283**: 2015–606.

65. Kermack KA, Mussett F and Rigney HW. The skull of *Morganucodon*. *Zool J Linn Soc* 1981; **71**: 1–158.

66. Sánchez‐Villagra MR, Gemballa S and Nummela S *et al.* Ontogenetic and phylogenetic transformations of the ear ossicles in marsupial mammals. *J Morphol* 2002; **251**: 219–38.

67. Clack JA and Allin EF. The evolution of single-and multiple-ossicle ears in fishes and tetrapods. In: Manley GA, Fay RR and Popper AN (eds.). *Evolution of the Vertebrate Auditory System Springer Handbook of Auditory Research*. New York: Springer, 2004, 128–63.

68. Kermack KA and Mussett F. The ear in mammal-like reptiles and early mammals. *Acta Palaeontol Pol* 1983; **28** 147–58.

69. Presley R. Lizards, mammals and the primitive tetrapod tympanic membrane. *Sym* *Zool Soc Lond* 1984; **52**: 127–52.

70. Mallo M. Formation of the middle ear: recent progress on the developmental and molecular mechanisms. *Dev Biol* 2001; **231**: 410–9.

71. O'Gorman S. Second branchial arch lineages of the middle ear of wild‐type and Hoxa2 mutant mice. *Dev Dynam* 2005; **234**: 124–31.

72. Takechi M, Kuratani S. History of studies on mammalian middle ear evolution: a comparative morphological and developmental biology perspective. *J Exp Zool Part B* 2010; **314**: 417–33.

73. McKenna MC, Kielan-Jaworowska Z and Meng J. Earliest eutherian mammal skull, from the Late Cretaceous [Coniacian] of Uzbekistan. *Acta Palaeontol Pol* 2000; **45**: 1–54.

74. Archibald JD and Averianov AO. Late Cretaceous asioryctitherian eutherian mammals from Uzbekistan and phylogenetic analysis of Asioryctitheria. *Acta Palaeontol Pol* 2006; **51**: 351–76.

75. Henson Jr OW. Comparative anatomy of the middle ear. In: Keidel WD and Neff WD (eds.). *The Handbook of Sensory Physiology: the Auditory System V/I*. New York: Springer-Verlag, 1974, 39–110.

76. Segall W. The auditory ossicles (malleus, incus) and their relationships to the tympanic: in marsupials. *Cells Tissues Organs* 1969; **73**: 176–91.

77. Koyabu D, Hosojima M and Endo H. Into the dark: patterns of middle ear adaptations in subterranean eulipotyphlan mammals. *Roy Soc Open Sci* 2017; **4**: 170608.

78. Zhang Z, Zhang X and Avniel WA *et al.* Malleal processus brevis is dispensable for normal hearing in mice. *Dev Dynam* 2003; **227**: 69–77.

79. Takeuchi Y, Hori M and Tada S *et al.* Acquisition of lateralized predation behavior associated with development of Mouth Asymmetry in a Lake Tanganyika Scale-Eating Cichlid Fish. *PloS one* 2016; **11**: e0147476.

80. Satokata I and Maas R. Msx1 deficient mice exhibit cleft palate and abnormalities of craniofacial and tooth development. *Nat Genet* 1994; **6**: 348–56.

81. Cockerell TDA, Miller LI and Printz M *et al.* The auditory ossicles of American rodents. *Bull Am Mus Nat Hist* 1914; **33**: 347–80.

82. Shute CCD. The evolution of the mammalian eardrum and tympanic cavity. *J Anat* 1956; **90**: 261–81.

83. Watson DMS and Wilson JT. VII. The monotreme skull: A contribution to mammalian morphogenesis. *Philos T R Soc Lond B, Containing Papers of a Biological Character* 1916; **CCVII.-B.341**: 335–47.

84. van Kampen PN. Die tympanalgegend des Säugetierschädels. *Gegenbaurs Morphol Jahrb* 1905; **34**: 321–722.

85. Macrini TE. Quantitative comparison of ontogenetic and phylogenetic character changes in the synapsid mandible and auditory region. *J Mamm Evol* 2002; **9**: 185–208.

86. Watson DMS. The evolution of the mammalian ear. *Evolution* 1953; **7**: 159–77.

87. Tucker AS, Watson RP and Lettice LA *et al.* Bapx1 regulates patterning in the middle ear: altered regulatory role in the transition from the proximal jaw during vertebrate evolution. *Development* 2004; **131**: 1235–45.

88. Maier W and Ruf I. The anterior process of the malleus in Cetartiodactyla. *J Anat* 2016; **228**: 313–23.

89. Anthwal N, Joshi L and Tucker AS. Evolution of the mammalian middle ear and jaw: adaptations and novel structures. *J Anat* 2013; **222**: 147–60.

90. Anthwal N, Urban DJ and Luo Z-X *et al.* Meckel’s cartilage breakdown offers clues to mammalian middle ear evolution. *Nat Ecol Evol* 2017; **1**: 0093.

91. Urban DJ, Anthwal N and Luo Z-X *et al.* A new developmental mechanism for the separation of the mammalian middle ear ossicles from the jaw. *P Roy Soc B: Bio* 2017; **284**: 20162416.

92. Hagenbach E. Ueber ein besonderes, mit dem Hammer der Säugethiere in Verbindung stehendes Knöchelchen. *Arch* *Anat Physiol Welt Med* 1841; **1841**: 46–54.

93. Mao F-Y and Meng J. Making a mammalian ear. Modular decoupling of the mammalian middle ear and jaw discovered in a new species of Cretaceous stem therian mammals. *Zoology* 2020; **140**: 125767.

94. Novacek MJ and Wyss A. Origin and transformation of the mammalian stapes. *Rocky Mt Geol* 1986; **24**: 35–53.

**Supplementary Figures**

**
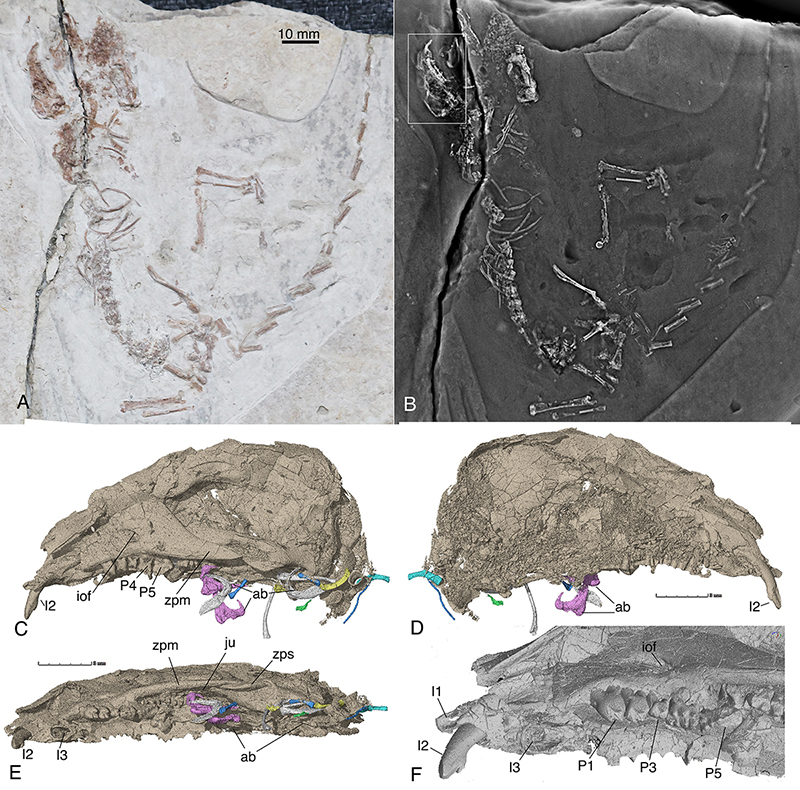
**

**Supplementary Figure 1.** **The holotype specimen of *Sinobaatar pani* sp. nov. (BPMC 0051).** A, Specimen in prepared condition. B, Computed laminographic image of the holotype skeleton. The boxed area is the partial skull shown in C-F. C, Lateral view of the left side of the skull preserved in the matrix. D, Exposed side of the broken skull. E, Ventral view of the crushed skull. F, Close-up of the partial skull in ventrolateral view. I1 and I3 were shown by alveoli. The colors of the auditory bones and hyoids correspond to those in Fig. 2, Supplementary Fig. 3 and Supplementary Fig. 5. Abbreviations: ab, auditory bones; I, upper incisor; iof, infraorbital foramen; ju, jugal bone; P, upper premolar; zpm, zygomatic process of maxilla; zps, zygomatic process of squamosal. See also Fig. 1 and Movie S1-3.


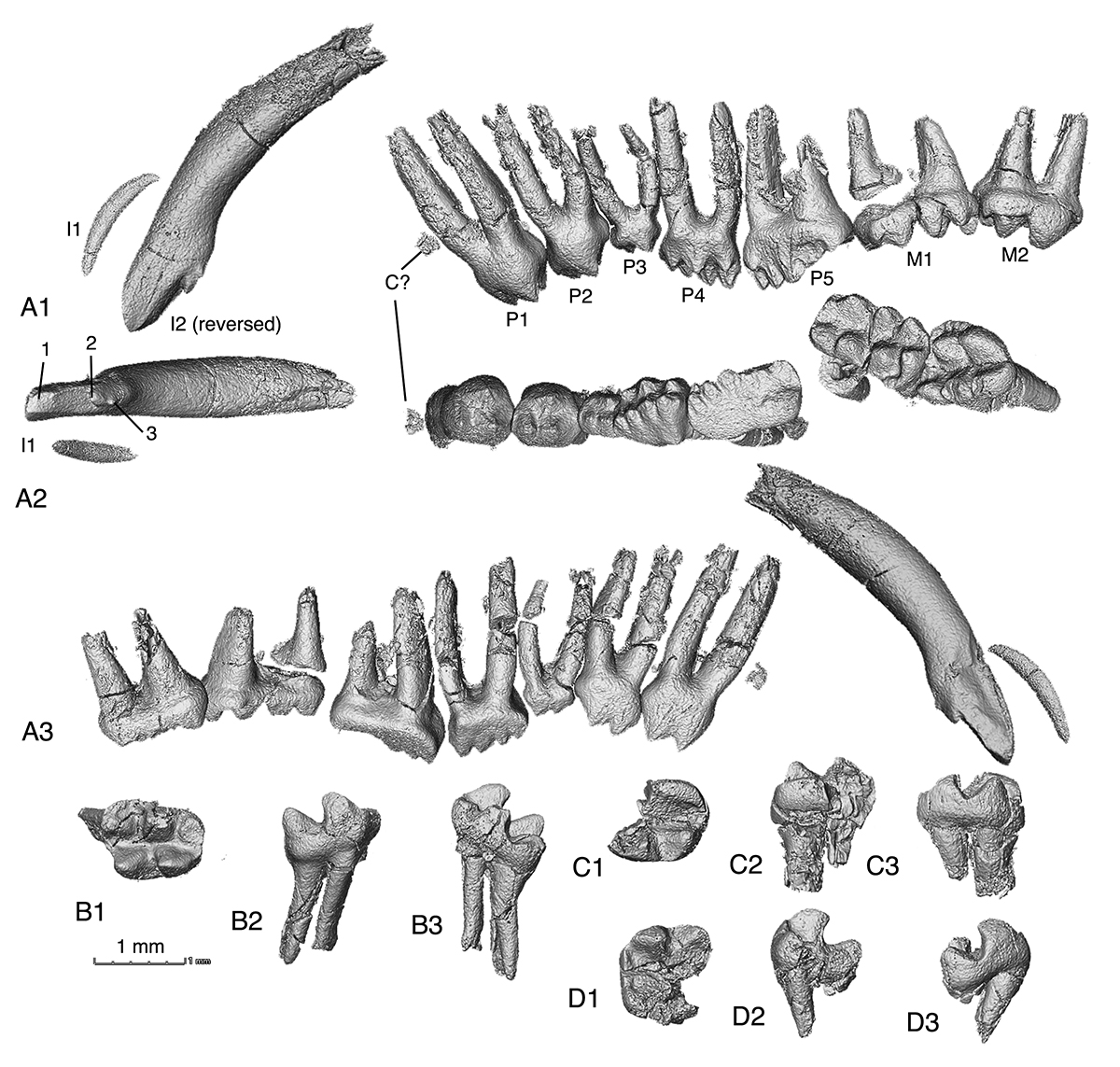


**Supplementary Figure 2.** **CT-rendered dentitions of *Sinobaatar pani* sp. nov. (BPMC 0051).** A1-3, Left upper dentition in labial, occlusal, and lingual views. B1-3, Left lower m1 in crown, lingual and labial views. C1-3, Partial left m2 in crown, lingual and labial views. D1-3, Partial right m2 in in crown, lingual and labial views. Compare the size difference with *Jeholbaatar* and *Sinobaatar*. Tooth measurements (length/width in mm): I1: 0.202/0.159; I2: 0.724/0.5; I3:?; P1:0.797/0.760; P2: 0.802/0.696; P3: 0.572/0.516; P4:0.874/0.787; P5:1.284/0.746; M1:1.378/0.842; M2: 1.114/0.983; m1: 1.121/0.905.


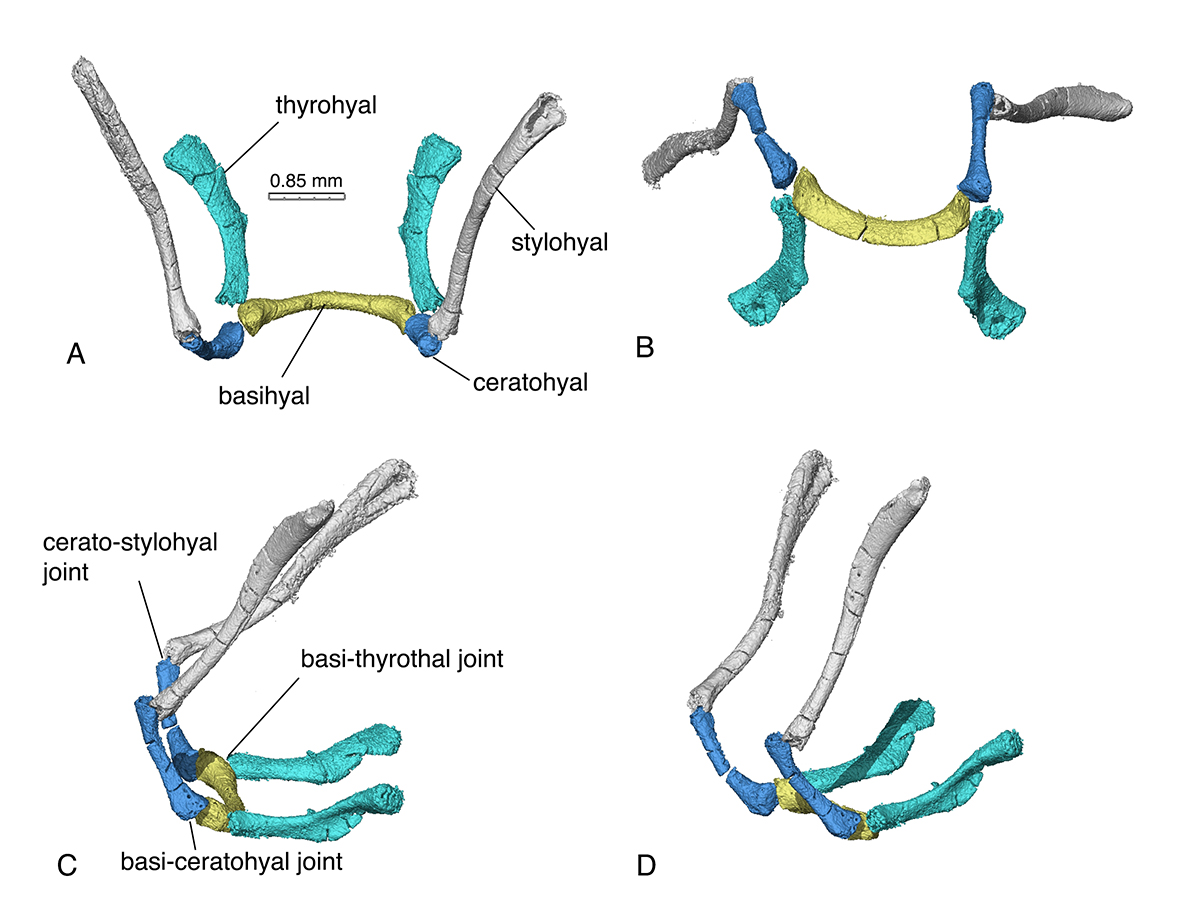


**Supplementary Figure 3.** **Digital rendered hyoid apparatus of *Sinobaatar pani* sp. nov*.* (BPMC 0051).** A, anterodorsal view; B, Ventral view; C, lateral view; D, anterolateral view. The right thyrohyal was not preserved and digitally flipped from the left one. Measurements (length in mm): Basihyal: 2.024; ceratohyal (left/right): 1.500/1.452; stylohyal (left/right): 3.562/3.676; thyrohyal (left): 1.993. See Supplementary movie 3.


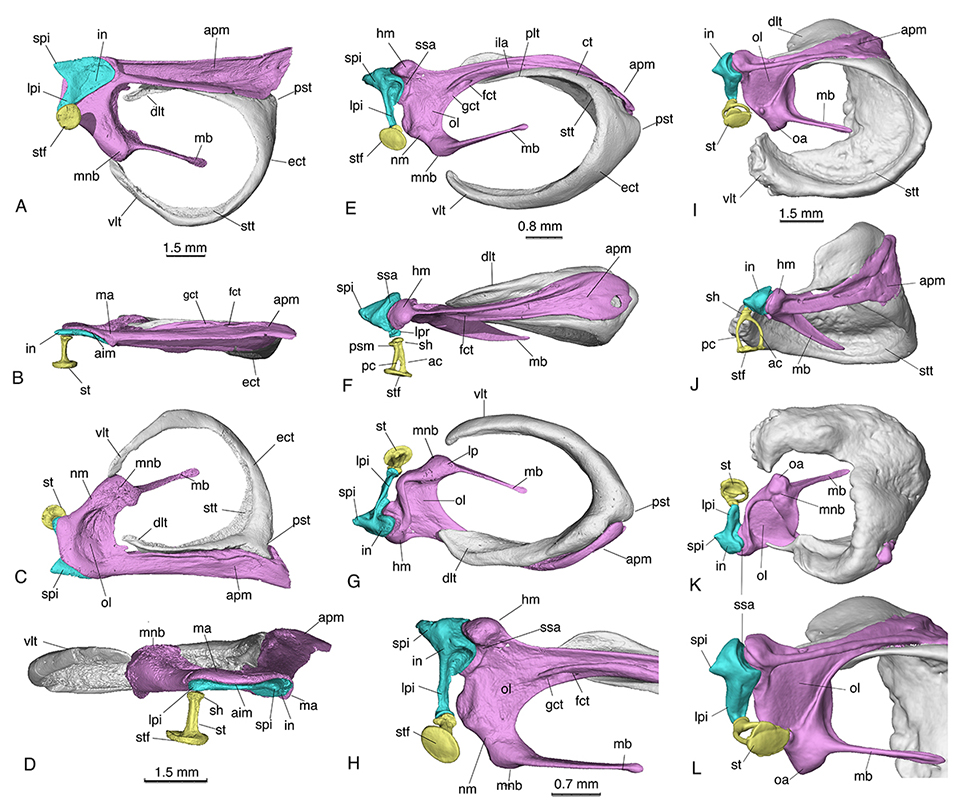


**Supplementary Figure 4.** **CT-rendered auditory bones of monotremes (*Tachyglossus*), marsupials (*Didelphis*), and placentals (*Erinaceus*).** A-C, Auditory bones of *Tachyglossus* in medial (dorsal), dorsal (lateral), and latera (ventral) views. D, Close-up posterior view of the auditory bones showing the flat contact between the malleus and incus. E-G, Auditory bones of *Didelphis* in corresponding views of A-C. H, Close-up view (more or less in medial) of auditory bones of *Didelphis*, showing the interlocking joint between the malleus and incus. I-K, Auditory bones of *Erinaceus* in corresponding views of A-C. All bones are in original articulation. Images are not on scale. Abbreviations: ac, anterior crus of stapes; aim, abutting contact of incus and malleus; apm, anterior process of malleus; ct, crista tympanica; dlt; dorsal limb of ectotympanic; ect, ectotympanic; fct, foramen for chorda tympani; gct, groove for chorda tympani; hm, head of malleus; ila, inner lamella; in, incus; lp, lateral process of malleus; lpi, long (stapedial) process of incus; lpr, lenticular process; ma, malleus part; mb, manubrium of malleus; mnb, manubrial base; nm, neck of malleus; oa, orbicular apophysis; ol, osseous lamina; pc, posterior crus of stapes; plt, posterior limb of ectotympanic bone; psm, process for stapedius muscle; pst, styliform process of tympanic bone; sh, stapedial head; spi, short process of incus; ssa, saddle-shaped articulation; st, stapes; stf, stapedial footplate; stt, sulcus tympanicus of tympanic bone; vlt, ventral limb of ectotympanic bone.


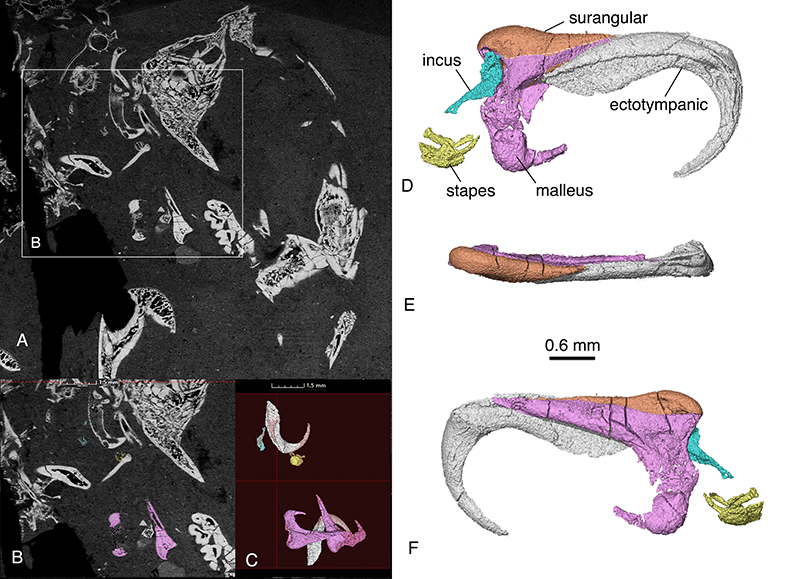


**Supplementary Figure 5. Preservation and auditory bones of *Sinobaatar pani* sp. nov. (BPMC 0051).** A, A slice of CT-scan imaging stack that shows the bone-matrix contrast and the auditory bones in original preservation. B, Auditory bones are high-lighted to show their positions in the scan slice. C, Auditory bones were segmented in their preserved positions. D-F, Reconstructed auditory bones in their interpreted anatomical positions; the relationship of the stapes is uncertain. The division of the fused malleus and surangular bodies is arbitrary, based on comparison with other forms (Supplementary Fig. 9). See Supplementary movie 4.


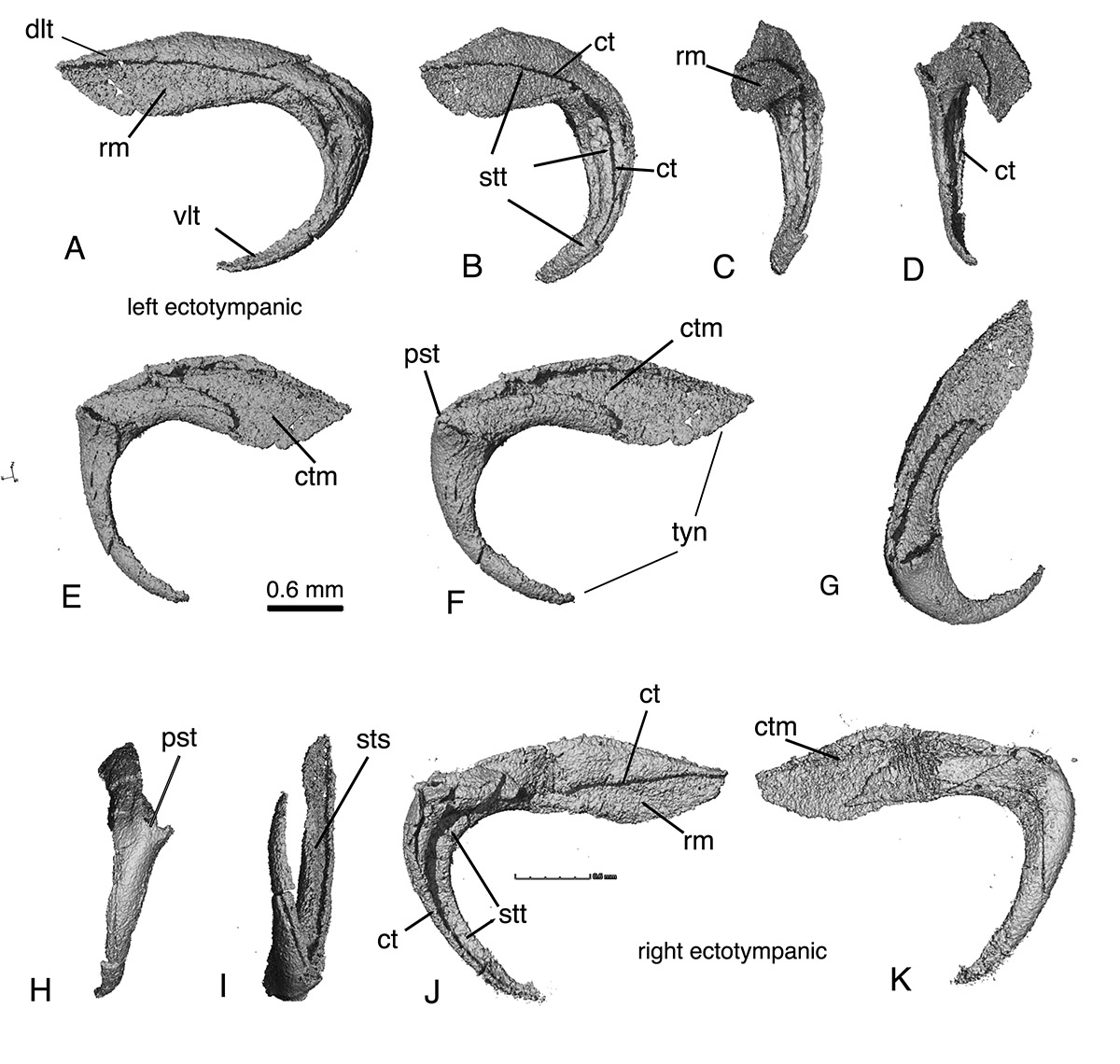


**Supplementary Figure 6.** **Ectotympanic (tympanic) bone of *Sinobaatar pani* sp. nov. (BPMC 0051).** A-I, Left ectotympanic in different views (A, medial; F, lateral). J-K, Right ectotympanic in medial and lateral views. Abbreviations: ct, crista tympanica; ctm, contact facet for malleus; dlt, dorsal limb of ectotympanic bone; pst, styliform process of ectotympanic bone; rm, recessus meatus; stt, sulcus tympanicus of tympanic bone; tyn, tympanic notch (incisure); vlt, ventral limb of ectotympanic bone.


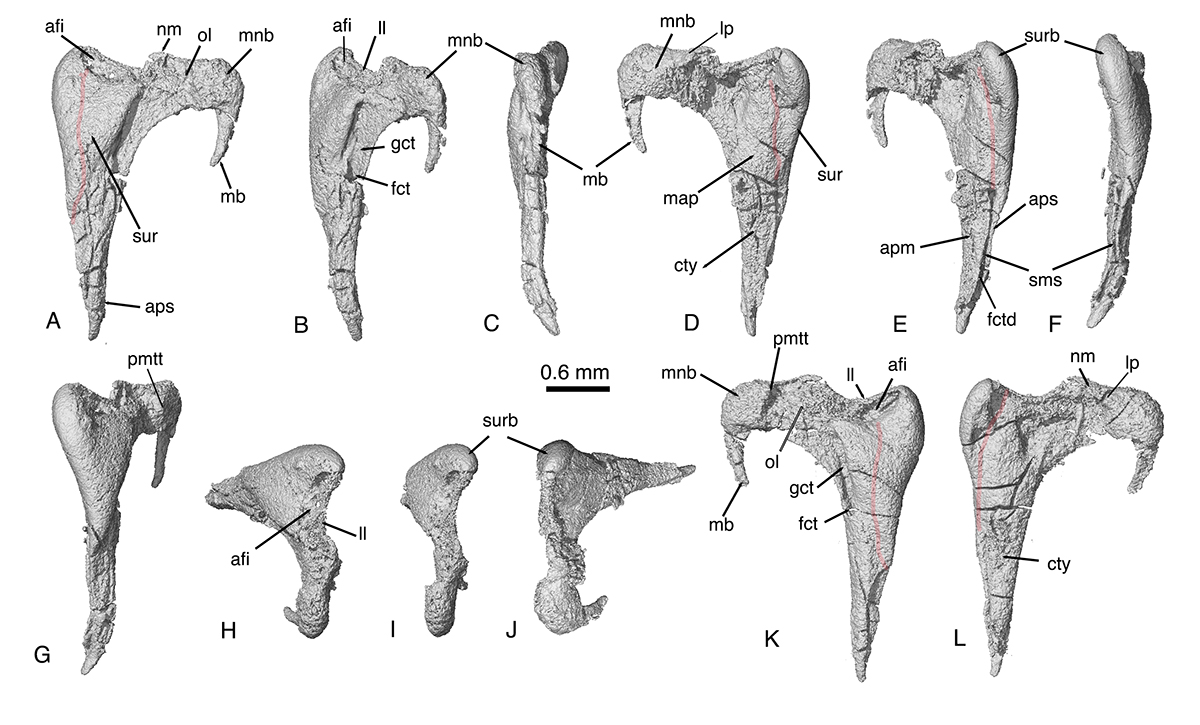


**Supplementary Figure 7.** **Malleus and surangular of *Sinobaatar pani* sp. nov*.* (BPMC 0051).** A-J, Right malleus in various views (A, medial; C, ventral; D, medial; F, dorsal; I, proximal). K, L, Medial and lateral views of the left malleus and surangular. Faint red line indicates the boundary between the malleus and surangular. Abbreviations: afi, articular facet for incus; apm, anterior process of malleus; aps, anterior process of surangular; cty, contact for ectotympanic; fct, foramen for chorda tympani; fctd, foramen (exit) for chorda tympani on dorsal side; gct, groove for chorda tympani; ll, lateral lip of the articular facet; lp, lateral process; map, malleus part; mb, manubrium; mnb, manubrial base; nm, neck of malleus; ol, osseous lamina; pmtt, muscular process for tensor tympani muscle; sms, suture between (anterior processes of) malleus and surangular; sur, surangular part; surb, surangular boss.


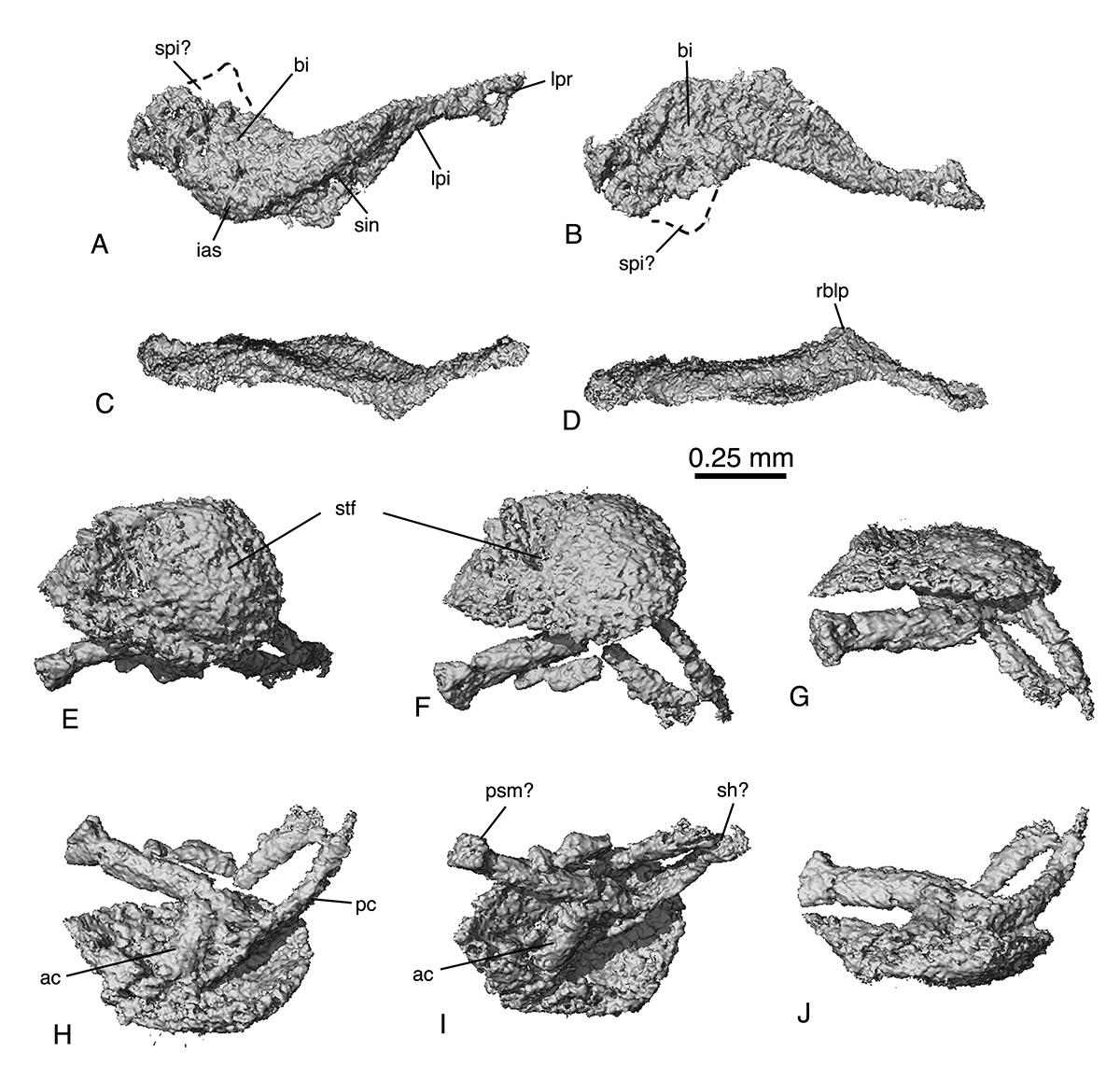


**Supplementary Figure 8.** **Incus and stapes of *Sinobaatar pani* sp. nov. (BPMC 0051).** A-D, Lateral, medial, anterior, and posterior views of the left incus. E-J, Various views of the stapes (presumably left) in which E is the proximal (medial) view (convex toward the fenestra vestibuli) and I is the lateral view. Abbreviations: ac, anterior crus of stapes; bi, body of incus; ias, incus articular surface for malleus; lpi, long (stapedial) process of incus; lpr, lenticular process; pc, posterior crus of stapes; psm?, process for stapedius muscle?; rblp, ridge between body and long process; sh?, stapedial head?; sin, sulcus incudes; spi, short process of incus (broken); stf, stapedial footplate.


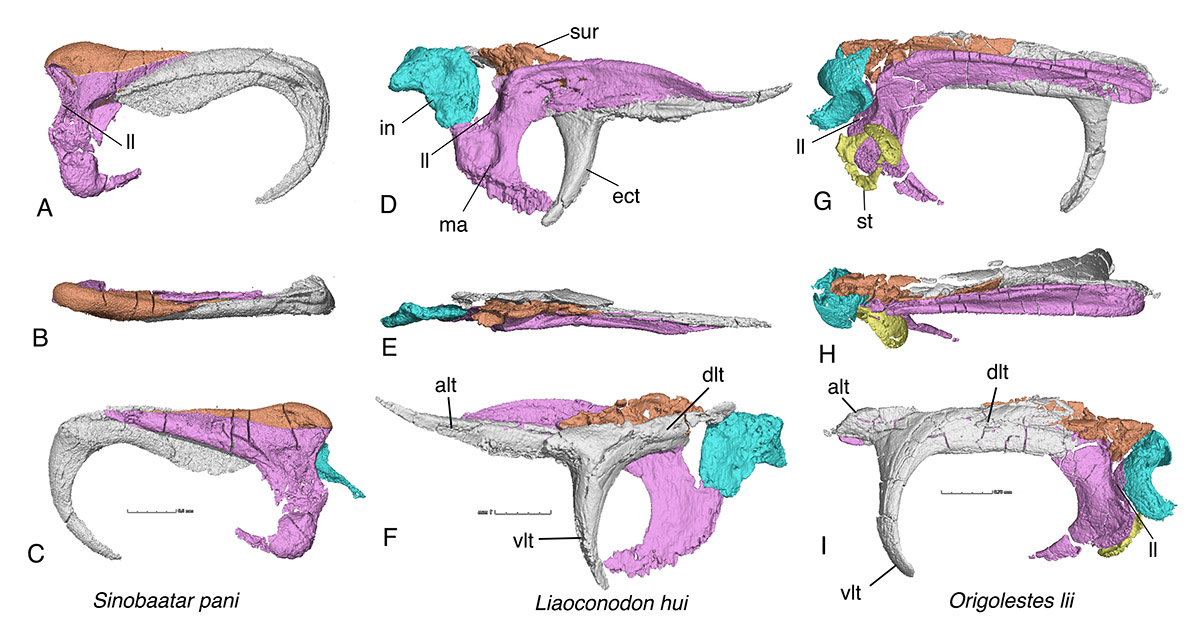


**Supplementary Figure 9. Comparison of auditory bones in *Liaoconodon*, *Oregolestes*, and *Sinobaatar* *pani* sp. nov.** A-C, Medial, dorsal and lateral views of the auditory bones of *S. pani* (the stapes is not included). D-F, Medial, dorsal and lateral views of the auditory bones of *Liaoconodon.* G-H, Medial, dorsal and lateral views of *Origolestes*. See Fig. 3 caption for the main differences among these forms. Abbreviations: alt, anterior limb of ectotympanic bone; dlt, dorsal limb of ectotympanic bone; ect, ectotympanic; in, incus; malleus part; ll, lateral lip of the articular facet; ma, malleus; st, stapes; sur, surangular part. Figures not to scale.

**Supplementary Movies**

Supplementary Movie 1. Preservation of the skull and auditory bones of *Sinobaatar pani* sp. nov.

Supplementary Movie 2. Reconstructed hyoids and auditory bones.

Supplementary Movie 3. Hyoid apparatus of *Sinobaatar pani* sp. nov*.*

Supplementary Movie 4. Auditory bones of *Sinobaatar pani* sp. nov.

Supplementary Movie 5. Auditory bones of *Liaoconodon*.

Supplementary Movie 6. Auditory bones of *Origolestes*.
